# Characterization of Novel Precursor miRNAs Using Next Generation Sequencing and Prediction of miRNA Targets in Atlantic Halibut

**DOI:** 10.1371/journal.pone.0061378

**Published:** 2013-04-23

**Authors:** Teshome Tilahun Bizuayehu, Jorge M. O. Fernandes, Steinar D. Johansen, Igor Babiak

**Affiliations:** 1 Faculty of Biosciences and Aquaculture, University of Nordland, Bodø, Norway; 2 Department of Medical Biology, Faculty of Health Sciences, University of Tromsø, Tromsø, Norway; Virginia Tech, United States of America

## Abstract

**Background:**

microRNAs (miRNAs) are implicated in regulation of many cellular processes. miRNAs are processed to their mature functional form in a step-wise manner by multiple proteins and cofactors in the nucleus and cytoplasm. Many miRNAs are conserved across vertebrates. Mature miRNAs have recently been characterized in Atlantic halibut (*Hippoglossus hippoglossus* L.). The aim of this study was to identify and characterize precursor miRNA (pre-miRNAs) and miRNA targets in this non-model flatfish. Discovery of miRNA precursor forms and targets in non-model organisms is difficult because of limited source information available. Therefore, we have developed a methodology to overcome this limitation.

**Methods:**

Genomic DNA and small transcriptome of Atlantic halibut were sequenced using Roche 454 pyrosequencing and SOLiD next generation sequencing (NGS), respectively. Identified pre- miRNAs were further validated with reverse–transcription PCR. miRNA targets were identified using miRanda and RNAhybrid target prediction tools using sequences from public databases. Some of miRNA targets were also identified using RACE-PCR. miRNA binding sites were validated with luciferase assay using the RTS34st cell line.

**Results:**

We obtained more than 1.3 M and 92 M sequence reads from 454 genomic DNA sequencing and SOLiD small RNA sequencing, respectively. We identified 34 known and 9 novel pre-miRNAs. We predicted a number of miRNA target genes involved in various biological pathways. miR-24 binding to kisspeptin 1 receptor-2 (*kiss1-r2*) was confirmed using luciferase assay.

**Conclusion:**

This study demonstrates that identification of conserved and novel pre-miRNAs in a non-model vertebrate lacking substantial genomic resources can be performed by combining different next generation sequencing technologies. Our results indicate a wide conservation of miRNA precursors and involvement of miRNA in multiple regulatory pathways, and provide resources for further research on miRNA in non-model animals.

## Introduction

MicroRNAs (miRNAs) are small (∼22 nucleotides/nts) non-coding RNAs, involved primarily in post-transcriptional regulation of mRNA when loaded onto RNA-induced silencing complex (RISC) [1]. They have been implicated in various physiological processes. Abrupt expression of miRNAs causes physiological disturbances, such as developmental defects and diseases [2], [3]. A miRNA gene can reside in an intergenic region, in an intron of a gene, or in a transposon region. miRNA suppression is mainly accomplished through the interaction of complementary sites of miRNA with that of 3′ UTR of a target mRNA, perhaps rarely with 5′ UTR and coding regions as well. In the RISC, a miRNA serves as a gene-specific guide that tethers the functional components of the complex to a target. In the canonical miRNA-mRNA interaction model, 5′ seed sequence (2–8 nt from the 5′ end) is the main target recognition site of a miRNA and has a role in linking the presumed target to Argonaute (Ago) protein complex. However, alternative non-canonical models of miRNA-mRNA interaction have been reported [4], [5], [6], [7].

Different algorithms have been developed for miRNA target prediction. Some of these prediction tools, such as RNAhybrid [8], miRanda [9], TargetScan [10], or PicTar [11] use preset criteria, such as sequence complementarity of the miRNA seed region, thermodynamic stability of mRNA:miRNA duplex, target site conservation among closely related species or other features, to identify the putative miRNA targets. Others tools, such as TargetBoost [12] or RNA22 [9], use machine learning algorithms to set the criteria. Both approaches have strengths, weaknesses, and high error rates, since miRNAs have diverse mechanisms of action and do not strictly follow a rule of thumb. The major use of virtual prediction tools is in identifying putative target genes for further experimental validation. Most algorithms are in searchable databases and contain only predictions for known miRNAs of a model organism. In addition, 3′ UTR sequence data for non-model species are limited, which is the major limitation in determining miRNA targets in these species. Thus, target prediction using publicly available sequences of a non-model species serves rather as a starting point for further experimental validation and contributes to comparison of miRNA functions across various species.

*In vivo* and *in vitro* miRNA target validations have been performed using various methods, including Ago-immunoprecipitation followed by sequencing, simultaneous miRNA and miRNA target expression analysis, RACE-PCR using mature miRNA as a primer, luciferase reporter assay, or miRNA overexpression or knockdown, amongst others [6], [7], [13], [14], [15].

To better understand regulatory roles of miRNAs during early ontogeny and sexual development in Atlantic halibut (*Hippoglossus hippoglossus*), a flatfish that undergoes dramatic metamorphosis events during the early development, we had previously generated miRNA libraries from various stages of development, and immature adult male and female brain and gonad. We have shown differential expression of various miRNAs during the major developmental transitions [16] and their sexually dimorphic expression during sexual development [17]. In this study, we generated a partial sequence of the Atlantic halibut genome DNA at an average of about 1× coverage using Roche 454 pyrosequencing technology. By combining genomic data with SOLiD small transcriptome data, we identified a number of conserved and novel pre-miRNAs, and validated them by RT-PCR. We also predicted a number of miRNA target genes from the Atlantic halibut EST database. This study confirmed the evolutionary conservation of many pre-miRNAs and the involvement of miRNA in many regulatory pathways.

## Materials and Methods

All Atlantic halibut husbandry and experimental procedures were performed in accordance with the Norwegian Regulation on Animal Experimentation (The Norwegian Animal Protection Act, No. 73 of 20 December 1974) and were approved by the National Animal Research Authority (Utvalg for forsøk med dyr, forsøksdyrutvalget, Norway) General License for Fish Maintenance and Breeding (Godkjenning av avdeling for forsøksdyr) no. 17. To collect tissues, animals were anesthetized with 1% MS-222 (3-aminobenzoate methanesulfonic acid, Sigma Aldrich) and sacrificed thereafter according to standard procedure.

### Genomic data acquisition

Atlantic halibut genomic DNA was extracted from ovarian tissue of a 5-years-old individual using MagMAX™ DNA Multi-Sample Kit (AB Applied Biosystems, Foster City, California, USA) and sequenced using Roche 454 GS FLX titanium sequencer, according to the manufacturer's instructions. The genomic DNA sequence was examined using FastQC (http://www.bioinformatics.babraham.ac.uk/projects/fastqc/) and *de novo* assembled using CLC (CLC Genomics Workbench 4.9).

### Discovery of miRNA precursors and mapping SOLiD reads

**Figure 1:**
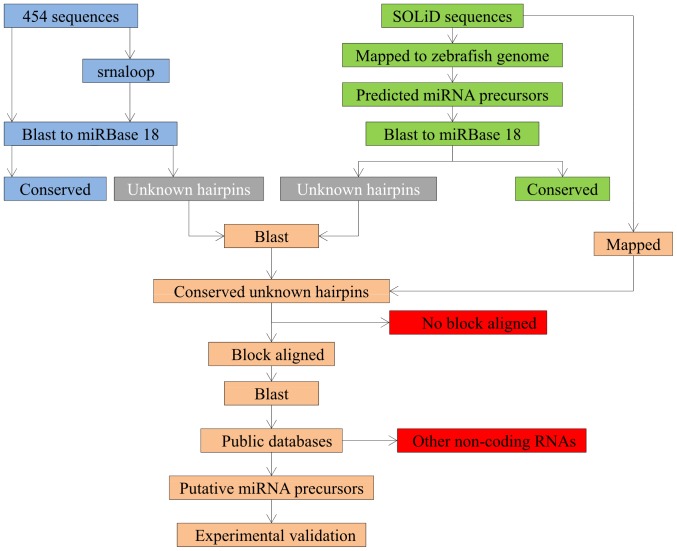
Data analysis strategy. The procedure is described in Materials and Methods.

We used small RNA transcriptome data generated previously using Sequencing by Oligonucleotide Ligation and Detection (SOLiD) [16], [17]. Data analysis strategy is shown in Fig. 1. Atlantic halibut SOLiD sequence reads were mapped to zebrafish genome sequences (Zv9.62) and hairpins were extracted using wapRNA tools in default setting [18]. miRBase 18 hairpin sequences were mapped to the 454 sequences to identify conserved pre-miRNAs. Novel pre-miRNAs were identified by extracting hairpins from 454 reads using srnaloop [19] using the parameters described previously [16], and mapping them to miRBase 18 (http://www.mirbase.org/) using CLC. Assuming the average length of fish pre-miRNAs and mature miRNAs at ∼86 nts and ∼22 nts, respectively, and taking into account non-conserved region of pre-miRNA, mapping was performed with 80% similarity in half of the length with 2 mismatches and 3 indels costs. All positively mapped sequences were checked manually and tandem repeat sequences were removed. The two mapping results were merged and redundant sequences were not included in further analysis. SOLiD small RNA sequence reads were mapped back to the identified conserved pre-miRNAs. The mapping was checked for block-like alignment outside the loop region of a putative pre-miRNA. Sequences fulfilling the above criteria were considered as known pre-miRNAs, whereas the rest of unmapped hairpins were mapped to putative pre-miRNAs obtained from zebrafish genome. Sequences with significant matches were blasted to the NCBI database, checked for similarity to other non-coding RNAs (rRNA, snoRNA, tRNA, snRNA), and removed when a match was found. The unmapped contigs were filtered using the same criteria for conserved pre-miRNAs as mentioned above and miRBase guidelines for miRNA annotation [20] All sequences were deposited in miRBase database (www.mirbase.org).

### Precursor identification using RT-PCR

Based on the results obtained from bioinformatics analysis, PCR primers were designed to amplify pre-miRNAs. Total RNA from various developmental stages: blastula, 50% epiboly, 20 somites, hatching, first feeding and pre metamorphosis, and various adult tissues: brain, skin, kidney, gut, ovary, testis, and liver was pooled and run on a 12% denaturing PAGE gel. The size fraction smaller than 200 bp was excised and eluted. Reverse transcription was performed on the size-fractionated RNA using SuperScript Vilo (Invitrogen, Carlsbad, CA, USA). RT-PCR was performed using initial denaturation step for 2 min at 94°C, followed by 25 cycles of 94°C for 15 s, 57–60°C for 20 s and 72°C for 15 s, with final extension time of 10 min at 72°C (Table 1).

**Table 1:** Primers used for RT-PCR and corresponding annealing temperatures (°C).

Label	Forward primer (5'–3')	Reverse primer (5'–3')	(°C)
mir-n1	TGTTTGACATGTTTATTGTCACCA	TTCAGATGTGTGAAGGCTGTC	59
mir-n3	TGCTTCCCCTTTTTCTGTTG	TTCTTTTCTTTCCTCCTGCTG	58
mir-n4	TTTCCTACTGTTTCCTTGATTTCTT	CTCTTTCTTCCACCCAACCA	57
mir-n5	GCACGGATAAAGGCAGTTTT	GCAAGCAAGGATAAGCACAG	58
mir-n6	ACTGGGGGCCAGACTGTACT	CGTGGCTGCCAGTTAAGTT	58
mir-n8	CACACATTCACTCGTGTCCA	CATGGGCAAACACATGTAGG	59
mir-n9	GAGCTACTGCGGGTGGTTTA	CACAGGCTTCTTCTGCATCA	60
mir-n10	GCCATGTTTGCTCTGAGTGA	GATCCGGTGTTTGAATGGAT	59
mir-n13	TCTTCAGTGGGTCAGTCATCC	CCAAACGGTACTCACTGTCCA	60
mir-n18	CACTGCAGAGTGAATGTGCTC	TGTACCCTACAAAGACAATGTGC	58
mir-n19	ACGGATCCAGAGGTGTGAAA	TGGATCAGAACCAGGTGTAGAA	60
mir-n20	GCTGGAAGGCAGTGTCTTGT	CAGCAGGTAAGCTGGCACTC	60
mir-n21	CAGCAGGTAAGCTGGCACTC	GCTGGAAGGCAGTGTCTTGT	60
mir-n22	TGCACAAGCACCAACTTGA	TCAATACTACCCCAACAAATAGCA	58
mir-n24	AGGAGACACGTGATGCTCTG	GGAGACTTATTCTGCTCATATCCA	58
mir-n26	GGTTTGTCCTCAGTGTTTTTGG	TTAGCAAGTGACGTCTTTAGAGC	58
mir-n29	CATAATTGACAGCGGGTCTG	CACTGGTTAGCTCGGGTCTG	60
mir-n30	GGCCATACCATCCTGAACAC	GACCCTGGTTAGTTTCCGAGA	60

### Computational target identification

3′ UTRs of Atlantic halibut genes were extracted from the NCBI database (March 17, 2012, http://www.ncbi.nlm.nih.gov/). These sequences were blasted against teleost database using 80% query coverage and identity with e-value <10^−5^. In addition, 3′UTRs of selected genes were cloned and sequenced using RACE (Invitrogen). RNAhybrid (v2.1) and miRanda (v3.3a) target prediction tools were used to identify putative miRNA target sites [8], [21]. To obtain a better estimate of statistical parameters from RNAhybrid, we performed a dinucleotide distribution of the 3′UTRs of halibut sequences using homerTools [22]. The RNAcalibrate option was used to generate 5000 random sequences with Gaussian distribution for accurate p-value calculation when obtaining the normalized minimum binding free energy. The miRNA:mRNA duplex formation was evaluated without constraint of seed nucleotide matching, since miRNA mechanisms of action could not strictly follow the seed pairing rule [6]. We used the consensus between the two prediction tools to increase the stringency, thereby reducing the number of false positive sequences [23].

Atlantic halibut genes were blasted and other teleost orthologs were retrieved from NCBI (downloaded 5 May 2012). The extracted 3′ UTR sequences were subjected to the same analysis as halibut 3′ UTR sequences as described above. The identified miRNA target sites were compared with those of Atlantic halibut. Predicted targets of Atlantic halibut miRNAs were classified into functional pathways using the Kyoto Encyclopedia of Genes and Genomes, KEGG [24].

Assuming that some miRNAs could have extensive sequence complementarities with their targets, we designed primers that matched miRNAs in question (miR-143, miR-202-3p, miR- 430, miR-733 and miR-2188), which were showing differential expression during early development and sexual development of Atlantic halibut [16], [17]. These primers were used to amplify possible targets using RACE-PCR. Then, the sequences were searched for miRNA target sites using the *in silico* method described above.

### Dual-luciferase reporter assay

The interaction between predicted targets and miRNAs was tested by designing sense and antisense oligonucleotides of the 3′UTR sequences of a target gene containing NheI and NotI restriction sites termini (Dataset S1A). Sense and antisense oligonucleotides were annealed as recommended by a manufacturer (Promega, Fitchburg, Wisconsin, USA). To construct pmirGLO-UTR, pmirGLO vector (Promega) was digested by NheI and NotI restriction enzymes and the annealed duplex was directionally cloned. For control, a scrambled sequence was constructed following the same procedure (Dataset S1A). Rainbow trout (*Oncorhynchus mykiss*) cell line RTS34st [25] was kindly provided by Prof. Peter Aleström, Norwegian School of Veterinary Science, Oslo, Norway. Cells were maintained in 70% Leibowitźs L15 medium (Sigma-Aldrich, St. Louis, Missouri, USA) supplemented with 30% fetal bovine serum (Invitrogen) and 0.5× antibiotics mix (Sigma-Aldrich): 2.5 mg/ml Amplicilin, 12 mg/ml Penicillin G and 20 mg/ml Streptomycin Sulphate. Lipofectamine 2000 (Invitrogen) was used for transfection. Transfectability of RTS34St cells was examined using pEGFP-N1 (Clontech Laboratories, inc, Mountain View, CA, USA). We transfected ∼10,000 cells per well in 96-well plates with a mixture including 100 ng pmirGLO-3′UTR construct and 30 nM of mirVana miRNA mimic (Life technologies); for controls, 100 ng of pmirGLO-3′UTR construct and pmirGLO-scramble were used. The dual-luciferase assays (Promega) were performed at 48 h after transfection and luminescence was measured using a BMG FLUOstar Optima (BMG Labtech GmbH, Offenburg, Germany). Statistical differences between treatment and control groups were determined using Student's t-test, at p<0.05.

## Results

### Conserved and novel pre-miRNAs of Atlantic halibut

**Figure 2:**
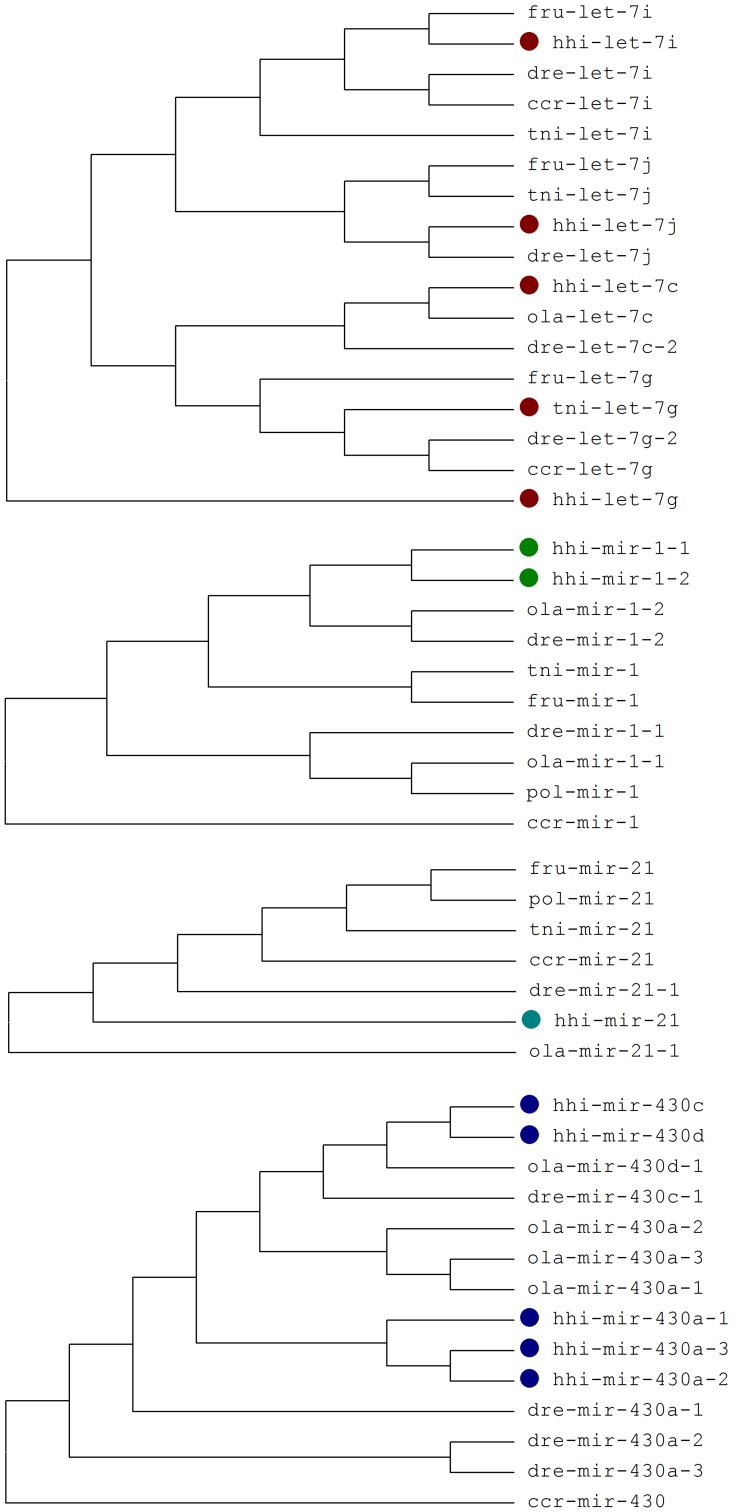
Phylogenetic trees for selected four miRNA families. The trees were constructed using maximum parsimony method using MEGA4. The precursor sequences for various teleost species were downloaded from miRBase. The abbreviations are: ccr (*Cyprinus carpio*); dre (*Danio rerio*), fru (*Fugu rubripes*); hhi (*Hippoglossus hippoglossus*); ola (*Oryzias latipes*), pol (*Paralichthys olivaceus*), and tni (*Tetraodon nigroviridis*).

We obtained more than 1.3 million sequence reads of Atlantic halibut genome, corresponding to approximately 1× coverage. *De novo* assembly resulted in 176976 contigs with N50 of 502 bp. We identified 29 pre-miRNAs and found additional 5 partial pre-miRNA sequences. The identified pre-miRNAs had similarities with pre-miRNAs of other teleosts (Fig. 2); mismatches were observed predominantly in the loop region and length differences also occurred (Dataset S2). We found pre-mir-196 and pre-mir-10 in the intergenic region of *hox* gene clusters (Dataset S1B). A cluster of miR-430 family was found both in the genomic 454 sequence data and in RACE-PCR transcript sequencing (Dataset S1C).

**Figure 3:**
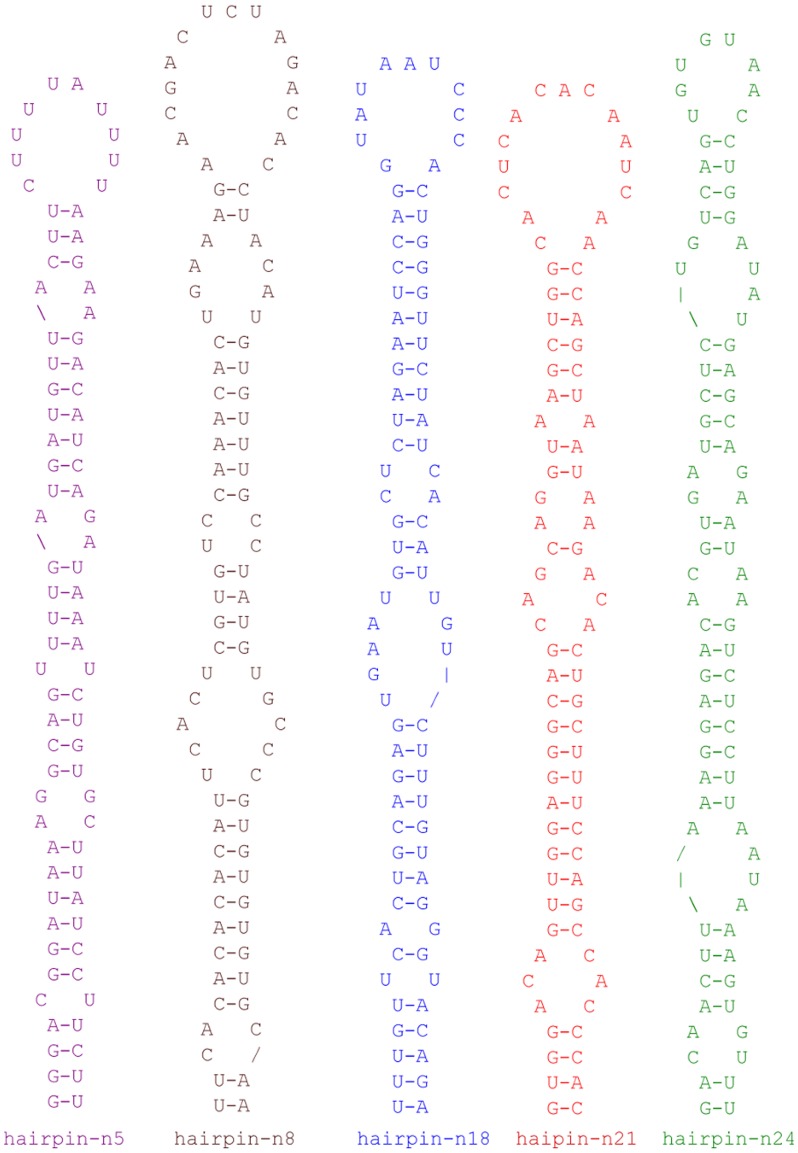
Predicted secondary structure of selected five miRNA hairpins from Atlantic halibut 454 genome sequences. For the secondary structure and corresponding free energy (ΔG in kcal/mol) of all predicted precursors, see Dataset S3.

**Figure 4:**
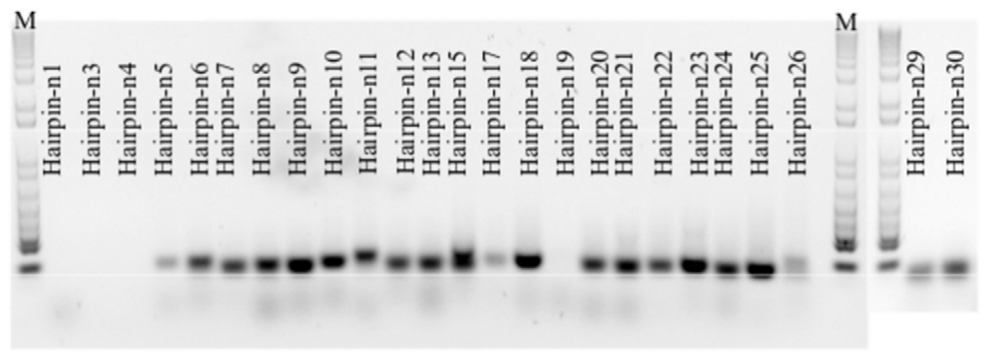
RT-PCR of Atlantic halibut novel hairpins. Small RNA fraction (<200 bp) was used to amplify the predicted precursor miRNAs. The name of each hairpin is assigned from n1 to n25. M stands for 1 Kb DNA size marker.

Twenty-five novel miRNAs were predicted from the 454 genomic sequences by their hairpin structure features and secondary structure free energy (Fig. 3 and Dataset S3). Two of the predicted hairpins (hairpin-n3 and -n30) were short with 54 bp and 57 bp, respectively. The free energy of these two hairpins was lower than in longer hairpins, such as hairpin-n7. However, small RNAs mapped to these hairpins had formed block-like alignments in at least one of the arms. Out of 25 predicted hairpins, 21 were amplified using RT-PCR of the small RNA fraction, <200 nts (Fig. 4).

### *In silico* target prediction

Partial or full 3′ UTR sequences were identified in 377 genes of Atlantic halibut downloaded from NCBI. Based on this dataset, the two target prediction tools, RNAhybrid and miRanda, predicted 45 and 92 possible targets for Atlantic halibut miRNAs, respectively (Table S1). Only 18 genes shared similar miRNA target sites between the two target prediction tools (Table S1). Twenty three Atlantic halibut genes shared homologous target sites with homolog genes of other teleosts.

Identified targets were involved in different biological processes according to KEGG classification (Table S1), including binding (20%), and catalytic and transporter molecules (6%). Some miRNAs were predicted to target metabolic pathways; for example, miR-301b was predicted to target 6-phosphogluconate dehydrogenase transcript. Some miRNAs were involved in multiple functional pathways, such as miR-1268 targeting multiple genes in binding, structural molecules and catalytic pathways (Table S1).

We found miRNAs targeting transcripts encoding cytokines: interleukin (*Il*)-*1β* targeted by miR-1957, *Il-6* by miR-1893, *Il-12β* by miR-24, and ubiquitin-like interferon-stimulated gene 15 (*Isg15*) by miR-214. Different transcripts of kinases were predicted as miRNA targets: lymphocyte-specific protein tyrosine kinase (*lck*) was targeted by miR-730 and miR-1249, and nucleoside diphosphate kinase by miR-1268 (Table S1). Neuroactive ligand receptor interaction genes transcripts, such as growth hormone receptor (*ghr*) and follicle stimulating hormone receptor (*fshr*) were targeted by miR-103 and miR-107, while luteinizing hormone receptor (*lhr*) and kisspeptin 1 receptor-2 (*kiss1-r2*) were targeted by miR-138 and miR-211, respectively. Hairy related 4 (*her4*) gene transcript was predicted by miRanda to be a target of many miRNAs including miR-138.

Some mRNAs had multiple miRNA binding sites; for example, *kiss1-r2* and interleukin-11b (*Il-11b*), having 2135 and 980 nts long 3′ UTRs, respectively, had 28 and 10 predicted miRNA binding sites, respectively. We tested whether the length of the 3′ UTR is correlated with the number of miRNA binding sites, but there were discrepant results between the two target prediction tools: a significant correlation between UTR length and number of miRNA binding sites was found in miRanda (ρ = 0.66, p<0.0001) but not in RNAHybrid (ρ = −0.14, p>0.05) analyses.

**Figure 5:**
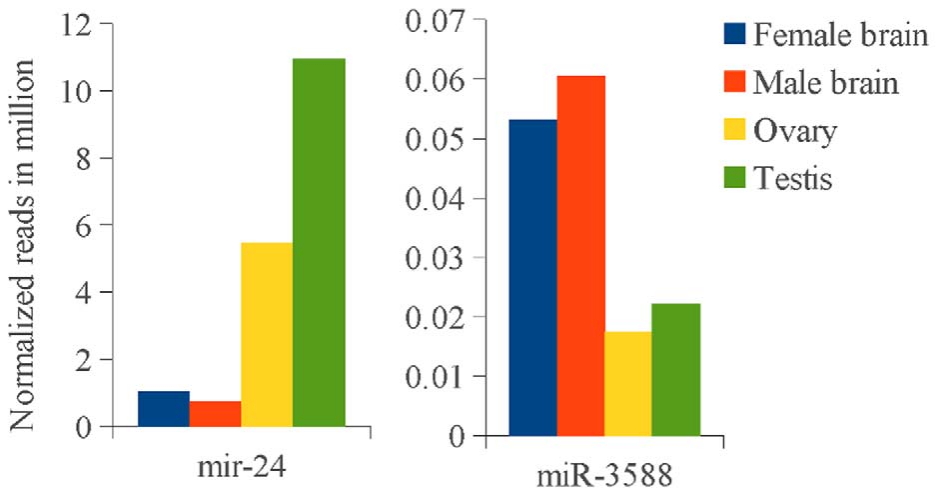
Expression of mir-24 and mir-3588 in brains and gonads. Normalized read count of 3 year-old immature Atlantic halibut female and male is obtained as the percentage of a miRNA count to the total miRNAs count in a given tissue, which is presented in millions.

Conservations of miRNA target sites were analyzed using ortholog genes from other teleosts. We found conserved miRNA target sites for some orthologs (Table S1). For example, miR-211 and miR-140 were predicted to target *ghr* product in Atlantic halibut, black seabream (*Acanthopagrus schlegeli*), fine flounder (*Paralichthys adspersus*) and Nile tilapia (*Oreochromis niloticus*); miR-1957 was predicted to target *Il-1β* in Atlantic halibut and plaice (*Pleuronectes platessa*); miR-148b was predicted to target cd3 gammadelta in Atlantic halibut and Japanese flounder (*Paralichthys olivaceus*); miR-143 was predicted to target elongation factor 1 alpha (*ef1a*) in Atlantic halibut and sea bream (*Sparus aurata*) and cobia (*Rachycentron canadum*). Cytochrome P450 aromatase brain isoform (*cyp19b*) was predicted to have a binding site for miR-3588 in Atlantic halibut and flathead grey mullet (*Mugil cephalus*). Relative frequency of miR-3588, which was calculated using the ratio of number of miRNA reads in a sample divided by the total number of miRNAs in the sample, was higher in brain compared to gonad (Fig. 5).

### Validation of some miRNA targets

**Figure 6:**
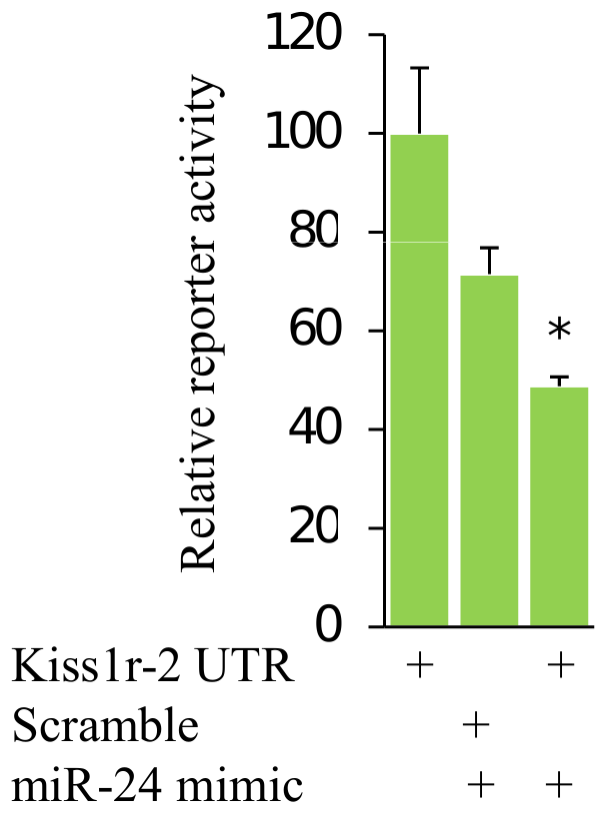
Repression of *kiss1r-2* by miR-24 in RTS34st cell line. The cells were transiently transfected with pmirGLO-kiss1r-2 vector construct or pmirGLO-kiss1r-2 vector construct together with miR-24 mimic or pmirGLO-scramble together with miR-24 mimic for 48 h followed by dual-luciferase reporter assay. The data represents average from two independent experiments each in three replicates, and are presented relatively to 3′ UTR of pmirGLO-*kiss1r-2*. Error bars indicate standard deviation and * shows significant (p<0.05) difference.

Amplification of possible targets of miR-143, miR-202-3p, miR-733, and miR-2188 using RACE-PCR was unsuccessful. We identified transcripts likely regulated by miR-430, which included collagen alpha-1 chain precursor, cytosolic nonspecific dipeptidase (*cndp2*), and osteonectin (*sparc*) (Dataset S4). Luciferase reporter assay demonstrated repression of *kiss1r-2* expression by miR-24 in RTS34st cell lines (Fig. 6). miR-24 was expressed abundantly in gonads as compared to brain (Fig. 5).

## Discussion

### Conservation of pre-miRNAs

The observed similarity of pre-miRNAs of Atlantic halibut and other vertebrates (Fig. 2 and Dataset S2) is in agreement with reports showing pre-miRNA conservation in the two arms but allowing variation in the termini and the big loop regions [26], [27], [28]. Some of the identified precursors were similar to mammalian pre-miRNAs but have not been previously reported in teleosts. As shown in several discovery and profiling studies in zebrafish [29], [30], [31], [32], a number of miRNA stills remains unidentified in teleosts.

The pre-miRNAs termini cut-off, used in this study, is based on the structure of hairpins. Although limitation of Atlantic halibut genomic resources have created a major challenge in demarcating the precursors termini, pre-miRNA size variation is suggested as a result of imperfect specificity of Drosha cleavage [33]. Thus, structural prediction may not clearly define the termini and the presented hairpin sequences may be subjected to addition or truncation of nucleotides.

The present results suggest the conserved arrangement of some miRNA genes in vertebrate genomes. For instance, miR-10 and miR-196 were found along with the *hox* gene cluster and similar arrangements have been reported in other teleosts [34] and mammals [35]. The associations of miR-430 family with satellite sequences also indicate the formation of miRNAs from repetitive elements of Atlantic halibut genome, as it has been demonstrated in other species [36].

### Targets of Atlantic halibut miRNAs

In the present study, miRNA targets prediction tools (RNAHybrid and miRanda) have shown differences in numbers and types of targets in Atlantic halibut miRNA binding site prediction. Employment of different prediction algorithms can reduce false positives, but increases false negatives [23], [37]. To reduce false positives many prediction algorithms use target sites conservation as a criterion [10], [37]; in this study, we found several conserved miRNA binding sites among teleosts. In addition, the correlation between the number of miRNA binding sites and the length of the 3′ UTR has shown discrepancy between the two prediction tools. Positive correlation between the length of the 3′ UTR and miRNA binding sites has previously been reported in mammals [38]. However, in our study, only miRanda algorithm showed such the correlation. The complexity of miRNA-mRNA interaction limits the reliability of prediction tools and there is no a toolkit prediction algorithm that can be used routinely. Thus, *in vivo* miRNA-mRNA interaction studies are crucial to investigate regulatory patterns and concede differences between prediction algorithms parameters.

The majority of Atlantic halibut ESTs are sequenced only from the 5′ end and do not contain 3′UTR sequences, which was the major limitation in predicting miRNA targets in this study. However, from the present data, the prediction tools together with gene ontology analysis of targets, suggest that Atlantic halibut miRNAs are involved in different biological pathways (Table S1). To which level these predictions are reliable, future experimental validation will disclose it. However, it is also possible to suggest that the predicted miRNA binding sites may be inaccessible for miRNA regulation, since some mRNAs possess secondary structures, which can protect against miRNA binding [39].

Genes involved in signaling pathways were among the predicted targets. Previously, the expression of cytokines under normal physiological conditions has been demonstrated in Atlantic halibut. For instance, *Il-1β* is expressed in various organs; *Il-12β* is highly expressed in immune related organs; and *Il-6* exhibits more limited expression pattern and is primarily detected in thymus and brain [40]. *Vibrio harveyi* challenge experiment in Asian seabass (*Lates calcarifer*) has shown rapid transient elevated expression of both *Il-1β* and miR-21 in three immune related organs, indicating role of miRNAs in acute inflammatory immune responses [28]. In mammals, miRNAs are reported to target cytokine transcripts [41], [42]. In this study, several miRNAs were predicted to target cytokines genes (Table S1), indicating that miRNAs can play a role in Atlantic halibut immune response.

Target site conservation across teleosts shows selective pressure. For instance, miR-3588, a member of the miR-125 family, was predicted to target brain aromatase (*cyp19b*) and the prediction was conserved across examined teleosts (Table S1). In the SOLiD data, we found higher number of miR-3588 reads in the brain as compared to gonads (Fig. 5). miR-125 is abundantly expressed in brain of teleosts [28], [43]. In addition, *kiss1r-2*, which is abundantly expressed in brain compared to gonad during Atlantic halibut maturation [44], is targeted by miR-34b and miR-574 (Table S1). In Atlantic halibut, miR-34b is expressed in brain, but not in gonad [17]. In addition, miR-24, which was predicted to target *kiss1r-2*, was abundantly expressed in Atlantic halibut testis (Fig. 5) [17]. *kiss1r-2* plays an essential role in the control of onset of puberty in vertebrates [2], [45]. The repression of *kiss1r-2* by miR-24 indicates a possible regulatory role of miRNA in sexual maturation (Fig. 6). Taken together, many miRNAs have conserved target genes among teleost and regulate multiple pathways.

The methodology used in this study demonstrated the considerable utility of NGS in deciphering and characterizing pre-miRNAs and miRNA target in animal species with limited genomic resources. The use of pathway models and other information from model species provide the possibility for functional annotation of genomic elements in non-model species. In addition, it allows extensive comparative studies. Thus, combining different sequencing technologies together with appropriate bioinformatics and available genomic resources can provide successful functional information on genetic elements of a non-model organism. Although SOLiD technology has been used in this study for deep sequencing, the proposed pipeline is versatile and other technologies, such as Illumina, could be applied as well.

In conclusion, alike mature miRNAs, many pre-miRNAs are conserved in Atlantic halibut in particular, and in teleosts in general. This sequence conservation is maintained to certain extent in target conservation among teleosts. Atlantic halibut miRNAs are predicted to target a number of genes involved in multiple biological pathways. Further 3′ UTR transcriptome sequencing, miRNA target site prediction and experimental validation are needed to decipher the biological relevance of miRNAs in teleosts.

## Supporting Information

Table S1 miRNA target prediction in Atlantic halibut using miRanda and RNAHybrid. Bold font indicates miRNA consensus between the two algorithms.
(XLS)

Dataset S1 A) 3'UTR oligo sequences; B) 454 genome sequence of the miR-430 cluster and pre-miR-10 and pre-miR-196; and C), RACE-PCR sequences of miR-430 transcripts from pooled samples of different developmental stages of Atlantic halibut (see Materials and Methods). Bold and highlighted letters indicate pre-miRNAs.
(PDF)

Dataset S2 pre-miRNA alignment. The conserved pre-miRNA sequence alignment (5′–3′) among teleosts. In cases of undiscovered miRNAs in teleosts, mammalian pre-miRNA orthologs were used. In addition to full Atlantic halibut precursor miRNAs, partial pre-miRNA sequences of Atlantic halibut are also aligned to teleosts ortholog. Highlighted bold sequences represent mature form of miRNA-5p; highlighted bold-italic represents mature miRNA-3p; and * stands for conserved nucleotide among species. The abbreviations are: ccr (*Cyprinus carpio*); dre (*Danio rerio*), fru (*Fugu rubripes*); hhi (*Hippoglossus hippoglossus*); hsa (*Homo sapiens*); mmu (*Mus musculus*); ola (*Oryzias latipes*), pol (*Paralichthys olivaceus*), ssc (*Sus scrofa*), and tni (*Tetraodon nigroviridis*).
(DOC)

Dataset S3 Hairpin structures predicted from Atlantic halibut sequenced genome data using srnaloop. The sequence centroid secondary structure and minimum free energy in kcal/mol are depicted.
(DOC)

Dataset S4 Amplified fragments of Atlantic halibut miR-430 target genes using RACE-PCR.
(PDF)
