# Supplementary material for: Characterization of Novel Precursor miRNAs Using Next Generation Sequencing and Prediction of miRNA Targets in Atlantic Halibut
Source: PLoS One. 2013 Apr 23;8(4):e61378. doi: 10.1371/journal.pone.0061378 (PMC3634072; doi:10.1371/journal.pone.0061378)
Supplement: Dataset S1 — A) 3'UTR oligo sequences; B) 454 genome sequence of the miR-430 cluster and pre-miR-10 and pre-miR-196; and C), RACE-PCR sequences of miR-430 transcripts from pooled samples of different developmental stages of Atlantic halibut (see Materials and Methods). Bold and highlighted letters indicate pre-miRNAs. (PDF) [file pone.0061378.s002.pdf]

Dataset S1

A)
> Kiss1r-2-sense
CTAGCTAGTTAACTAGTGCAACCCGCGTGGCCTGGGCCCGC

> Kiss1r-2-antisense
GGCCGCGGGCCCAGGCCACGCGGGTTGCACTAGTTAACTAG

> Scramble sequences
ATGGGCCCGCGGCCGCTCTAGAAGTACTCTCGAGAAGCTTTTTGAATTCTTTGGATCCACTAGTGTCGA
CCTGCAGGCGCGCGAGCTCCAGCTTTTGTTCCCTTTAGTGAGGGTTAATTTCGAGCTTGGCGTAATCAA
GGTCATAGCTGTTTCCTGTGTGAAATTGTTATCCGCTCACAATTCCACACAATATACGAGCCGGAAGTA
TAAAGTGTAAAGCCTGGGGTGCCTAATGAGTGAGCTAACTCACAGTAATTGCGG

B)
> miR-430 cluster (mir-430a-2, mir-430a-16, mir-430c-1)
TTATGGAAATCCAACTCTGATCACCTCAAATAGAGCCACTCATGATTACTGGCTTCATAAGTGCTTCTCT
TTGGGGTTGTCTTGGTTGGGATATTTTCTGCAATATAATATTTCAGTAATGATTCAATTCCAGTGAATGA
AATCAGACATCAATAGAAACTGCTATGTAGTTGGTTGTGTATCCAAAAATATTCAGCAAAATTTGAAAAT
AACTTTCAGAAGACAATTTTTTTAACAAAGCCTACATTTTGTTACACAACTAGGTG**GTCACCAGCTTTAC**
CCTAACACAAGCAAAGACTTGCCTTCTGAAAGCAGTAAGTGCTATTTGTTGGGGTAGTGTTGCTGATTTT
GCTATTCTTTTTCCTTCACACAGTTCATGAAGACAGACATGTTATATCATTAATTGCTGAAGAATTCCTA
GTTTGAGAGTT**GGTCCTACCTATTACCCTAACTTGAGCATTAACTTGTTTGTTGAATGCAGTAAGTGCTA**
CTTGTTGGGGTAGTTTTAAGTGACAACAACAACTGAACGCATCAAGAGCCAATTCCCCTCGT**GAATCCAA**
CTCAGATCACCTCAAATGGAGCCACTGATGATTCTCTTCTTCATAAGTGCTTCTCTTTGGGGTTGTCTTG
GTTGGGATAAGTATGCGATGGAAAGATCCCAGTCGAGCTGAATAAACAATCTCGAATTCATGTGTACAAT
CACGGGACATTTTCAGCAAGTAAGAGATGCTTTTCAGCTTAATTCACAAACACAGCCTCTCAGCTTCATT
TGCATACTAGAGGATAGCTCTTTATAGTTGGGATGTGATATTTTACTGAAGTACAACATAAATAGTGGTT
TAGGTTCATTGCTCTGTTAGGCATACAATTACACCCACTGCCTCAATTTTGGAGTAAGAATGCAGTGCTA
GTTGGTATTACTTTAGAAACTTCTTAGCTGCAACTGCAGGCT

>miR-430 cluster (mir-430c-2, mir-430a-1)
ACATGATAGTCGTCTCATGAAGGTGTGATCCTTTCCTTAACGAACCTTTACCAAGACCATTGAAACTTCT
TACTAAAAGAGGGTGGGGCAGGGTGGGTGTGGCTACATTCAAGTTTAAGATGGGCAACGTTGTAGAAGAC
AATCCACTGCTGCTGGAACACAACACAGAAGATGCTGCAAAGGACCATCTGGACCATGAGGCAAGTCTTA
ACTAAGGTAGAGTTTTTGTTAAAAAGGATTTTGCCAGTGGCCAAGCTCCAACACTGACTACTATATTATT
TCAGCAAATTGTTATGGTAGTTTTACTCGACAGACGCCATAACTTGAAGCTACATGAAGCTTAAATTAAA
TCATCTCAAATACTCACTTATGGTTCCTTTCTTGACAATTGTTTTTCTTTGTCTGTGTCCCTGGTTGTAA
GTTTTCAGAAAACCTGGTCACTGCCAGACTGTATTTTTTTTATTGTTAGCTAAGCTTATCTAACTTGTTG
GGGTAGTGTTGCTGATTTTGCAATTCTTTTTCCTCCACACAGTTCATGAATACACACATGTTACATCATT
AATTGCTGAAGAACTCCTCTGTCCTACCTATTACCCTAACTTGAGCATTAACTTGTTTGTTGAATGCAGT
AAGTGCTACTTGTTGGGGAAGTTTTAAGTGACAACAACAACTGAATGAATCAAAAGCCATTTCCCCTTGT
GAATCC**AGCTCAGATCACCTCAAATGGAGCCACTGATGATTCTCTTCTTCATAAGTGCTTCTCTTTGGGG**
TTGTCTTGGTTAAGTCAAGTTCTTCATTGGGGAAATCTAAACTGTACTGAGTAACAGTACAGAAATCACT
TGTCCACAAATGTGGAACAAATTTCAGGAAGACATACACTGTAGTTGAATAACATTCAAATGTGAGCAGA
AAAAAGCCCTACACAGTTTGGATGTTAGTGAATGGACTTAACTTCATGTATGTGAAGAATCTGCAGTTTG
TCCTAAAACATAAAATTGCAATTCTGACTAGCTAGATTC**ATCAATTTATCACCAATATTACCCTTGCACA**
AGCACCAACTTGAGTTTAGAAAACAGTAAGTGCTATTTGTTGGGGTAGTATTGATGATTTCAAATGTCCA
TGTTCTCTTCACAGCTGCTTTGACATCCATTTCTGGTGTTTTANGACTGGAAAGAACATTGC

>HoxAb gene (mir-196a)
TTTTGGACTTTTACAGGTTTCCAAATATGTAGCCAGATTTTAAACCAAGAAACTACAGTTTTCACACTAA
AACACGTTGCTAAAACACGTTGTGGCGGATTTTTGCAGAAGAGG**AGAGCGGACTGTTGAGTGGTTTAGGT**
AGTCTCATGTTGTTGGGCTAAATTATTTCTCCCACAACACGAAACTGCCTTGATTACCTCAGTAAAACTC
GTCGCCACAC

>HoxDb gene (mir-10d)
TGGATCCAGTGGCGTAATCACATTGTGTATGTCGGTGTGTGCAACCATCCGTTTGGGCGATCGCAGATTG
GAGCTTTTTACGTGCACAGCGTTGCACAAATGTCAGTCAGGTATACGGATTTGGAAATGAATGATTTTAA
AGAAGGGAGAGACGTGAACACATGCATGCAAGTCAGAGGCTGTGTGAATGTAAACAGTGCTGGGTTCTTC
CTCCCGTCCGTCCGTGGCTGTGATG**GATGGTTAGTCATCGCCCATAGACTCCCCTAGAACCCAATCTGTG**
ACCGAGGCATCTCCACACACATTCGGTTCTACAGGGTATATATAGGCGACGGCTCACTTCACGGCCGGCT
GCTGATGACTTCCTCTCGTCGTCGACAGAGATTACACGCGTGTCGTCGTTCACCGAGACGCGCACCTTCT
ATGTTCTCCACATAT

C)
> pri-mir-430
GTGCTATTTGTTGGGGTAGTGTTGCTGATTTTGCTATTCTTTTTCCTTCACACAGTTCATGAAGACAGACATGTTACATCATT
AATTGCTGAAGAATTCCTAGTTTGAGAGTTGGTCCTACCTATTACCCTAACTTGAGCATTAACTTGTTTGTTGAATGCAGTAA
GTGCTACTTGTTGGGGCAGTTTTAAGTGACAACAACTGAACGCATCAAGAGCCAATTCCCCTCGTGAATCCAGC**TCAGATCAC**
CTCAAATGGAGCCACTGATGATTCTCTTCTTCATAAGTGCTTCTCTTTGGGGTTGTCTTGGTTGGGATAAGTATGCGATGGAA
AGATCCCAATAGAGCTGAATAAACAATCTCGAATTCATGTGTACAATCACGGGACATTTTCAGCAAGTAAGAGATGCTTTTCA
GCTTAATTCACAAACACAGCCTCTCAGCTTCATTTGCATACTAGAGGATAGCTCTTTATAGTTGGGATGTGATATTTTACTGA
AGTACAACATAAATAGTGGTTTAGGTTCATTGCTCTGTTAGGCATACAATTACACCCACTGCCTCAATTTTGGAGTAAGAATG
CAGTGCTAGTTGGTATTACTTTAGAAACTTCTTTAGCTGCAACTGCAGGCTACAATCTGGCAATGAGATGCATTCACAACAGT
AGTTGGATTCCAATTGGCCTATCATCAACAACCCTGCATACGTGATAGTCGTCTCATGAAGGTGTGATCCTTTCCTTAACGAA
CCTTTACCAAGACCATTGAAACTTCTT

> pri-mir-430
AGTGCTATTTGTTGGGGTAGTGTTGCTGATTTTGCTATTCTTTTTCCTTCACACAGTTCATGAAGACAGACATGTTACATCAT
TAATTGCTGAAGAATTCCTAGTTTGAGAGTT**GGTCCTACCTATTACCCTAACTTGAGCATTAACTTGTTTGTTGAATGCAGTA**
AGTGCTACTTGTTGGGGTAGTTTTAAGTGACAACAACAACTGAACGCATCAAGAGCCATTTCCCCTTGTGAATCCAAC**TCAGA**
TCACCTCAAATGGAGCCACTGATGATTCTCTTCTTCATAAGTGCTTCTCTTTGGGGTTGTCTTGGTTGGGATAAGTATGCGAT
GGAAAGATCCCAGTCGAGCTGAATAAACAATCTCGAATTCATGTGTACAATCACGGGACATTTTCAGCAAGTAAGAGATGCTT
TTCAGCTTAATTCACAGACACAGCCTCTCAGCTTCATTTGCATACTAGAGGATAGCTCTTTATAGTTGGGATGTGATATTTTA
CTGAAGTACAACATAAATAGTGGTTTAGGTTCATTGCTCTGTTAGGCATACAATTACACCCACTGCCTCAATTTTGGAGTAAG
AATGCAGTGCTAGTTGGTATTACTTTAGAAACTTCTTTAGCTGCAACTGCAGGCTACTATCTGGCAATGAGATGCATTCACAA
CAGTAGTTGGATTCCAATTGGCCTATCATCAACAAACCCTGCATACATGATAGTCGTCTCATGAAGGTGTGATCCTTTCCTTA
ACGAACCTTTACCAAGACCATTGAAACTTCTTA

> pri-mir-430
GGTAAACGAAATTTCGCCCCTTGTAAGTGCTATTTGTTGGGGTAGTATTGATGATTTCAAATGTCCATGTTCTCTTCACAGCT
GCTTTGTACATCCATTTCTGGTGTTTTATGACTGGAAAGAACATTGCAAGTCTTTTGTAT**GTCTTTACTATTACCCTGACTTG**
AGTATTGACTTGACAGTTGAATTCAGTAAGTGCTACTTGTTGGGGCAGTTTTAATCGACAAAAACAACATGAAAAGACACAAT
GTTATGGAA**ATCCAACTCTGATCACCTCAAATAGAGCCACTCATGATTATTGGCTTCATAAGTGCTTCTCTTTGGGGTTGTCT**
TGGTTGGGATATTTTCTGCAATATAATATTTCAGTAATGATTCAATTCCAGTGAATGAAATCAGACATCAATAGAAACTGCTA
TGTAGTTGGTTGTGTATCCAAAAATATTCAGCAAAATTTGAAAATAACTTTCAGAAGACAATTTTTTTAACAAAGCCTACATT
TTGTTACACAACTAGGTG**GTCACCAGCTTTACCCTAACACAAGCAAAGACTTGCCTTCTGAAAGCAGTAAGTGCTATTTGTTG**
GGGTAGTGTTGCTGATTTTGCTATTCTTTTTCCTTCACACAGTTCATGAAGACAGACATGTTATATCATTAATTGCTGAAGAA
TTCCTAGTTTGAGAGTT**GGTCCTACCTATTACCCTAACTTGAGCATTAACTTGTTTGTTGAATGCAGTAAGTGCTACTTGTTG**
GGGTAGTTTTAAGTGACAACAACAACTGAACGCATCAAGAGCCAATTCACCTCGTGAATCCA**ACTCAGATCACCTCAAATGGA**
GCCACTGATGATTCTCTTCTTCATAAGTGCTTCTCTTTGGGGTTGTCTTGGTTGGGATAAGTATGCGATGGAAAGATCCCA
