# Supplementary material for: Characterization of Novel Precursor miRNAs Using Next Generation Sequencing and Prediction of miRNA Targets in Atlantic Halibut
Source: PLoS One. 2013 Apr 23;8(4):e61378. doi: 10.1371/journal.pone.0061378 (PMC3634072; doi:10.1371/journal.pone.0061378)
Supplement: Dataset S2 — pre-miRNA alignment. The conserved pre-miRNA sequence alignment (5′–3′) among teleosts. In cases of undiscovered miRNAs in teleosts, mammalian pre-miRNA orthologs were used. In addition to full Atlantic halibut precursor miRNAs, partial pre-miRNA sequences of Atlantic halibut are also aligned to teleosts ortholog. Highlighted bold sequences represent mature form of miRNA-5p; highlighted bold-italic represents mature miRNA-3p; and * stands for conserved nucleotide among species. The abbreviations are: ccr (Cyprinus carpio); dre (Danio rerio), fru (Fugu rubripes); hhi (Hippoglossus hippoglossus); hsa (Homo sapiens); mmu (Mus musculus); ola (Oryzias latipes), pol (Paralichthys olivaceus), ssc (Sus scrofa), and tni (Tetraodon nigroviridis). (DOC) [file pone.0061378.s003.doc]

**Dataset S2**

hhi-let-7c ------AUGUGUGCAUCCGGGU**UGAGGUAGUAGGUUGUAUGGUU**UAGAAUUACACCCUGGGAGUUAACUGUACAACCUUCUAGCUUUCCUUGGAGUACACGU-----

ola-let-7c ------AUGUGUGCAUCCGGGU**UGAGGUAGUAGGUUGUAUGGU**UUAGAAUUACACCCUGGGAGUUAACUGUACAACCUUCUAGCUUUCCUUGGAGCGCACGU-----

dre-let-7c-2 --------GUGUGCAUCCAGGC**UGAGGUAGUAGGUUGUAUGGUU**UCGAAUGACACCAUGGGAGUUAACUGUACAACCUUCUAGCUUUCCUUGGAGUACAC-------

****** ** *********************** ** * ** ************************************** ***

hhi-let-7j GGUC**UGAGGUAGUUGUUUGUACAGUU**UG-AGGGUCUGUGAUUCUGCCCCAUACAGGAGCUAACUGUACAAGUGACUG-------

fru-let-7j -GUC**UGAGGUAGUUUUUUGUACAGUU**UG-AGGGUCUGUGAUUCUGCCCCAUACAGGAGCUAACUGUACAAGUGACUGCCUUGCC

tni-let-7j -GUC**UGAGGUAGUUUUUUGUACAGUU**UG-AGGGUCUGUGAUUCUGCCCCAUACAGGAGCUAACUGUACAAGUGACUGCCUUGCC

dre-let-7j -GGU**UGAGGUAGUUGUUUGUACAGUU**UUUAGGGUCUGUUAUUCUGCCCUGUUAAGGAGCUAACUGUACAGACUACUGCCUUGCC

* ********** ************ ********* ********* * **************** ****

hhi-mir-1 ----AAGAUUACCUCCUUGGUGCACAUACUUCUUUAUGUACCCAUAUGAACAUAUGAUAGCUA**UGGAAUGUAAAGAAGUAUGUAU**UCCUGGUGAGGUGCG--------

tni-mir-1 -------AUUACCUCCUUAGUGUACAUACUUCUUUAUGUACCCAUAUGAACAUAUGAUAACUA**UGGAAUGUAAAGAAGUAUGUAU**UCUUGGUGAGGUGA---------

fru-mir-1 ---------------CUUAGUGUACAUACUUCUUUAUGUACCCAUAUGGACAUAUGAUAACUA**UGGAAUGUAAAGAAGUAUGUAU**UCUUGG-----------------

dre-mir-1-1 GCCCAUA-UAUCCCGCUUGGUAGACAUACUUCUUUAUAUGCCCAUAUGAACAAG-AGCAGCUA**UGGAAUGUAAAGAAGUAUGUAU**CCCAGGUGAGAGAAAAACGGGGC

ola-mir-1-1 ---GACAGCGGCCUGCUUGGGGGACAUACUUCUUUAUAUGCCCAUAUGAACACG-AGCAACUA**UGGAAUGUAAAGAAGUAUGUA**UCCCAGGUU-GGGAGAAAUGAC--

ccr-mir-1 ---------UGCCUACUUGGUGUACAUACUUCUUUAUGUGCCCAUAUGAACAUAUGACAGCUA**UGGAAUGUAAAGAAGUAUGUAU**UCUUGGUCAGGU-----------

pol-mir-1 -----------------------**ACAUACUUCUUUAUAUGCCCAUA**UGAACAAG-AGCAACUA***UGGAAUGUAAAGAAGUAUGUAU***-----------------------

*** * ************** * ******** *** * *************************

hhi-mir-10d -----UGAAGUGAGCCGUCGCCUAUAUA**UACCCUGUAGAACCGAAUGUGUGUG**GAGAUGCCUCGGUCACAGAUUGGGUUCUAGGGGAGUCUAUGGGCGAUGACUAACCAUC----------

ola-mir-10d ------GACGGGAACCUUCGCCCAUACA**UACCCUGUAGAACCGAAUGUGU**GUGGAGCUGCCUCAGUCACAGAUUGGGUUCUAGGGGAGUCUAUGGGCGAUGACUAACCAUC----------

fru-mir-10d -CCGGUGAGGUGGAUCGUCGUCUAUAAA**UACCCUGUAGAACCGAAUGUGUGUG**CAGCUGACUUGAUCACAGAUUGGGUUCUAGGGGAGUCUAUGGGCGAUGAAUAAUCACUGA--------

tni-mir-10d GCCGGUGAGGUGC-UCGUCGUCUAUACA**UACCCUGUAGAACCGAAUGUGUGUG**CAGCUGACUUGAUCACAGAUUGGGUUCUAGGGGAGUCUAUGGGCGCUGAAUAAUCAUCGAUGAACGGC

dre-mir-10d -UGGAAGCUUUGUUCCGUCGUCUAUAUA**UACCCUGUAGAACCGAAUGUGUG**UUUACACAGCAAAUUCACAGAUUCGGUUUUAGGGGAGUAUAUGGACGAUGCAAAAACGUCUGCUUUCA--

ccr-mir-10d -----CUCUUUGUUCCGUCGUCUAUAUA**UACCCUGUAGAACCGAAUGUGU**GUUUACACAGCACAUUCACAGAUUCGGUUUUAGGGGAGUAUAUGGACGAUGCAAA----------------

* * * *** * *** ************************* * * ********* **** ********* ***** ** ** *

hhi-mir-15a ----GACAGCAGAUCUGGUGAUGCUG**UAGCAGCACGGAAUGGUUUGUG**GGUUGUA--------------------------------------------------

fru-mir-15a --------------CUGGUGAUGCUG**UAGCAGCACGGAAUGGUUUGUG**GGUUACA-CUGAGAUACAGGCCAUACUGUGCUGCCGCA-------------------

tni-mir-15a --------------CUGGUGAUGCUG**UAGCAGCACGGAAUGGUUUGUG**AGUUACA-CUGAGAUACAAGCCAUGCUGUGCUGCCGCA-------------------

dre-mir-15a-1 -------------CCUGUCGGUACUG**UAGCAGCACAGAAUGGUUUGUG**AGUUAUAACGGGGGUGCAGGCCGUACUGUGCUGCGGCAACAACGACAGG--------

ola-mir-15a -GCUGACCGGAGCUCUGGUGAUGCUG**UAGCAGCACGGAAUGGUUUG**UGGGUUAUUGUGAGAUGCAGGCCAUGCCGUGCUGCCGCAGUAAUGCCCGAUUACACAAC

ccr-mir-15a-1 ----------------------GGUG**UAGCAGCACAGAAUGGUUUG**UGGUGAUACAGAGAUGCAGGCCGUGAUGUGCUGCAGCAUC

*********** ************

hhi-mir-21 -----------------------------------CCAUCCCGUCAGC**UAGCUUAUCAGACUGGUGUUGGC**UGUUGUAUUGCCAUGGCAACACUAGUUUGUAAGCUGGCUGAAGCUGUGGGCCU

tni-mir-21 ---------AUUGUCGUCUUU-ACAUCUGUCACUCUCGGCCUGUCAAA**UAGCUUAUCAGACUGGUGUUGGC**UGUUAAGAUUGCAAGGCGACAACAGUCUGAAGGCUGUCUGACAUUUCGGGCUC

fru-mir-21 -----------------------------------------UGUCAAA**UAGCUUAUCAGACUGGUGUUGGC**UGUUAAGAUUGCAAGGCGACAACAGUCUGUAGGCUGUCUGACA----------

dre-mir-21-1 UUAUGUGUCUUUAUUGGCGUGGAUAUAAGUCUUUCCCAGUGUGUCAGA**UAGCUUAUCAGACUGGUGUUGGC**UGUUACAUUCGCCCGGCGACAACAGUCUGUAGGCUGUCUGACAUUUUGGGCAU

ola-mir-21-1 -----------------------------ACUUCACCACCUUGUCGGC**UAGCUUAUCAGACUGGUGUUG**GAGGUUCCAUUUGCACGGCAACAACGGUCUGUAAGCUGGCUGACAUUGUGGGGCU

ccr-mir-21 ---------------------------------UCCCAUUCUGUCAGG**UAGCUUAUCAGACUGGUGUUGGC**UGUAAGUCAUAU--GGCAACAUCAGUCUAAUAGGCUGCCUGAAAUUUUGGGCU

pol-mir-21 ------------------------------------------------**UAGCUUAUCAGACUGGUGUUGGC**UGUUUAGAUUGCAAGG***CGACAACAGUCUGAAGGCUGUC***---------------

********************** ** *** ** *** * *

hhi-mir-21 ------------------------

tni-mir-21 UUUCAUCU----------------

fru-mir-21 ------------------------

dre-mir-21-1 UUUCUUCUCCGAUUAAAAAUAUGA

ola-mir-21-1 U-----------------------

ccr-mir-21 ------------------------

pol-mir-21 ------------------------

hhi-mir-23a -GUUGUCUCUGUUGACCAGGGGAAUUCCUGGCAGGGUGAUUUUUGAGAC---UACAGGACUGA**AUCACAUUGCCAGGGAUUUCCA**AUGGCUGACAUG---

fru-mir-23a-1 -------GCUGU-GGCGGGGAGGGUUCCUGGCACCGUGAUUUGAUGCACAAAGACAAACAAAA**AUCACAUUGCCAGGGAUUUCCA**CCCUUUCACAG----

tni-mir-23a-1 -------GCUGU-GGCGGGGAGGGUUCCUGGCACCGUGAUUUGAUGCACAAAGACAAACAAAA**AUCACAUUGCCAGGGAUUUCCA**CCCUUUCACAG----

dre-mir-23a-1 -------GCUGU-GGCGGGGAGGGUUCCUGGCACCGUGAUUUGGUGGAUAAACAGAAAUGAAA**AUCACAUUGCCAGGGAUUUCCA**CUCCUGCACGGU---

ola-mir-23a-1 AUUUGAGUCUGUCGGCCAGGGGAAUUCCUGGCAGAGUGAUUUUUGAGAC---UACAGGACUGA**AUCACAUUGCCAGGGAUUUCCA**AUGGCUGCCAUGAAU

ccr-mir-23a -----------CCAGCUGGAGGGAUUCCUGGCAGAGUGAUUUGGGAUUA-----UAUCAUAAA**AUCACAUUGCCAGGGAUUUCC**AACCAGCU--------

* * * ********* ******* ***********************

hhi-mir-26-1 ------UUCGCUGUAACCUGG**UUCAAGUAAUCCAGGAUAGGC**UUUCUGUAUCUGCUUCGGCCUAUGCUUGAUUACUUGCACUUGGAGGCAGCAACAA-----

fru-mir-26 -------AGGCCUCGGCCUGG**UUCAAGUAAUCCAGGAUAGGC**UGGUUAACCCUG-CACGGCCUAUUCUUGAUUACUUGUGUCAGGAAGUGGCCGUG------

tni-mir-26 --GCGUUAGGCCUCGGCCUGG**UUCAAGUAAUCCAGGAUAGGC**UGGUUAACCCUG-CACGGCCUAUUCUUGAUUACUUGUGUCAGGAAGUGGCCGCCAGC---

ola-mir-26-3 UGCGUUGUUGCUGGAACCAGG**UUCAAGUAAUCCAGGAUAGGC**UUUCUGCAUCUGAUUUGGCCUAUACUUGAUUACUUGCACUUGGAACCAGUUCCUGACCCA

** ** ************************ * *** ******* ************ *** *

hhi-mir-26-2 ----CAGGGCCUGGGUCUGG**UUCAAGUAAUCCAGGAUAGGCU**UGUUUUAGUGGGGGAAGCCUAUUCAGGACGACUUGGUUCAGAAACAAGGCCG----

dre-mir-26a-1 ----------UUUGGCCUGG**UUCAAGUAAUCCAGGAUAGGCU**UGUGAU-GUCCGGAAAGCCUAUUCGGGAUGACUUGGUUCAGGAAUGA---------

ola-mir-26-1 GUCUCUGGGCCUCUGCCUGG**UUCAAGUAAUCCAGGAUAGGC**UGGUUAACACUGGCACGGCCUAUUCUUGAUUACUUGUUUCAGGAACCGGCCAUAAGC

ccr-mir-26a ----AGUGGCUGUUCCCUUG**UUCAAGUAAUCCAGGAUAGGCU**GUCU--GUCCUGGGAGGCCUAUUCAUGAUUACUUGCACUAGGUGGCAGCCGUUU----

************************** ** * ******** ** ***** **

hhi-mir-96 ----------------UGCCCAU**UUUGGCACUAGCACUAUUUUUGCU**UUUGUUCUCGUACUUUGAGCAAUCAUGUGUACGUGCCAAUAUCGGACGA-------------------------------

tni-mir-96 ---------------UUGCCCAU**UUUGGCACUAGCAC-AUUUUUGCU**UCUGUAUAUAUACUUUGAGCAAUUAUGUGUA-GUGCCAAUAUAGGAGA--------------------------------

fru-mir-96 --------CUCUUCUUCGCCCAU**UUUGGCACUAGCAC-AUUUUUGCU**UCUGUAUGUAUACUUUGAGCAAUUAUGUGUA-GUGCCAAUAUAGGAGAAGACAGACUUUCAACCU---------------

dre-mir-96 GCUGGGCGCUCUUCUUUGCCUGU**UUUGGCACUAGCAC-AUUUUUGCU**UUUUUAUAUAUACCUUGAGCAAUUAUGUGUA-GUGCCAAUAUGGGACAAGACAGACAUGCUACUUAAAAAAAAAAUCAGC

ccr-mir-96 --------CUCUUCUUUGCCUGU**UUUGGCACUAGCAC-AUUUUUGCU**UUUUUAUAUAUACGCUGAGCAAUCAUGUGUA-GUGCCAAUAUGGGACAAGACAGAAAU-----------------------

*** *************** ********** * * *** ********* ******* ********** ***

hhi-mir-129-1 ----UUCACGAAU**CUUUUUGCGGUCUGGGCUUGCU**GUUCAUAACUAUUAAUCUGGGAAGCCCUUACCCCAAAAAGCAUUUGCGGAGGGCGCA

fru-mir-129-2 --------UGGGU**CUUUUUGCGGUCUGGGCUUGCU**GUUCCUAAGGCAGUAGCCAGGAAGCCCUUACCCCAAAAAGUAUCUG-----------

tni-mir-129-2 ---------GGGU**CUUUUUGCGGUCUGGGCUUGCU**GUUCCUAAGGCAGUAGCCAGGAAGCCCUUACCCCAAAAAGUAUCU------------

dre-mir-129-2 GUCUUUCACGAAU**CUUUUUGCGGUCUGGGCUUGCU**GUUCUCAACUAUCAAU--GGGA***AGCCCUUACCCCAAAAAGCAU***UUGCGGAGGGC---

* *************************** ** * ********************* ** *

hhi-mir-129-2 ----------GUCCUUUGUGGGU**CUUUUUGCGGUCUGGGCUUGCU**GUUCCUAAGGCAGUAG-CC***AGGAAGCCUUAACCCCAAAAA***CGUAAUCUGCACUGGA------

fru-mir-129-1 ----------GUCCUUCACGAAU**CUUUUUGCGGUCUGGGCUUGCU**GUUCUUUAC-UGGUAAUCUGGGAAGCCCUUACCCCAAAAAGC--AUUUGCGGAGGAC-----

tni-mir-129-1 ----------GUCCUUCACGAAU**CUUUUUGCGGUCUGGGCUUGCU**GUUCUCUGC-UCA----CCCGGAAGCCCUUACCCCAAAAAGC--AUUUGCGGGGGGC-----

dre-mir-129-1 ----------GUCCUUUUCAGGU**CUUUUUGCGGUCUGGGCUUGCU**GUUCCUUGAACCAGUAGCCAGGAAGCCCUUACCCCAAAAAGU—AUCUGCAGAGGAC------

ccr-mir-129 UCCCUGUCUUGUCCUUUUCAGGU**CUUUUUGCGGUCUGGGCUUGC**UGUUCCUUGAACCAGUAGCCAGGAAGCCCUUACCCCAAAAAGUAUCUGCAGAGGACCAAAACA

****** *************************** * ******* * ********** ** *** **

hhi-mir-181b AGGAGGCGUUCACUCUCAAAGGCUGCAAUA**AACAUUCAUUGCUGUCGGUGGGUU**UACA-UAAGAAUAACUCACUGAUCAAUGAAUGCAGACUGCGGUUCAAACACCUCAGUAA

fru-mir-181b-2 --------------------GCUCGCAAUA**AACAUUCAUUGCUGUCGGUGGG**UUUCUA-UGGCAAUAGCUCACUGACCAAUGAAUGAAGACUGCGG-----------------

tni-mir-181b-2 --------------------GCUCGCAAUA**AACAUUCAUUGCUGUCGGUGGG**UUUCCA-UGGCAAUAGCUCGCUGACCAAUGAAUGAAGACUGCGG-----------------

dre-mir-181b-2 ---------------CUAAUGACUGCAAUA**AACAUUCAUUGCUGUCGGUGGG**UUUCUAAUAGACACAACUCACUGAUCAAUGAAUGCAAACUGCGGUGCAA------------

ola-mir-181b-2 UCCAGGAGUCAAC--CUUAAGGCUGCAAUA**AACAUUCAUUGCUGUCGGUGGGUU**UAUG-CAGGAACAACUCACUGAUCAAUGAAUGCAAACUGCGGUUCGCACAUCCUGCUGA

ccr-mir-181b -----------------AAUGACUGCAAUA**AACAUUCAUUGCUGUCGGUGG**GUUUUAAAUUGACACAACUCACUGAUCAAUGAAUGCAAACUGCGGUGCA-------------

* ******************************* * * *** **** ********* * *******

hhi-mir-182 -----CUCUCUGGUGGUG**UUUGGCAAUGGUAGAACUCACACUG**GUGAGGUAGAUGGAUCCGG***UGGUUCUAGACUUGCCAACUACUGC***CUGAGAGCGUCGAC

ola-mir-182 UUUGGUUCUCUAGUGGUG**UUUGGCAAUGGUAGAACUCACAC**UGGUGAGGUAGAUGGAUCCGGUGGUUCUAGACUUGCCAACUACUGCUUGAGAGUGUCCAA

fru-mir-182 ----------------UGUUUGGCAAUGGUAGAACUCACACUGGUGAGGUAGAUGGAUCCGG**UGGUUCUAGACUUGCCAACUA**CUGCU-------------

tni-mir-182 ----------------UGUUUGGCAAUGGUAGAACUCACACUGGUGAGGUAGAUGGAUCCGG**UGGUUCUAGACUUGCCAACUA**------------------

dre-mir-182 ---------------GUA**UUUGGCAAUGGUAGAACUCACA**CUGGUGAGGUAGUCAGAUCCGG***UGGUUCUAGACUUGCCAACUA***------------------

ccr-mir-182 -AAGGUUCUCCGAGGGUA**UUUGGCAAUGGUAGAACUCACAC**UGGUGAGGUAGUCAGAUCCG***GUGGUUCUAGACUUGCCAACUA***CUACCUGAGAACA-----

pol-mir-182 ------------------**UUUGGCAAUGGUAGAACUCACA**CUGGUGAGGUAGAUGGAUCCGG***UGGUUCUAGACUUGCCAACUA***------------------

* ********************************** ****************************

hhi-mir-183 CCUCAUAUCUCCUCCUGUUCUGUG**UAUGGCACUGGUAGAAUUCACUGU**CACAGCACACAAUCAGUGAAUUACCAUAGGGCCAUAAACAGAGUAGAGACAGAUCC-----

dre-mir-183 ---------GACUCCUGUUCUGUG**UAUGGCACUGGUAGAAUUCACUG**UGAAAGCACACUAUCAGUGAAUUACCAAAGGGCCAUAAACAGAGCAGAGAAAGAACCACG--

tni-mir-183 -------------------CUGUG**UAUGGCACUGGUAGAAUUCACUG**UGAGAGCUCACUAUCAGUGAAUUACCAUAGGGCCAUAAACAG--------------------

fru-mir-183 -------------------CUGUG**UAUGGCACUGGUAGAAUUCACUG**UGAGAGCUCACUAUCAGUGAAUUACCAUAGGGCCAUAAACAG--------------------

ola-mir-183-1 AUCCACAAUUCCUCCUAUUCUGUGU**AUGGCACUGGUAGAAUUCA**CUGUCACAGCACUCUAUCA***GUGAAUUACCAUAGGGCCAUA***AACAGAGUAGAGACAGAACCUCAGU

ccr-mir-183 ---------------UGUUCUGUG**UAUGGCACUGGUAGAAUUCACUG**UGAAAGCACACUAUCAGUGAAUUACCAAAGGGCCAUAAACAGAGCAGA--------------

***************************** * *** * * *************** **************

hhi-mir-187 GUGACCUCUCUGGCCGGGCCAAGGGCUGCAACACAGGACAUGGGUCCUGCU-CUCCUCCCCGC***UCGUGUCUUGUGUUGCAGCC***AGUGGAGCUGCCUG-----

ola-mir-187 ----------UGGUUGGGCCAGCGGCUGCAACACAGGACAUGGGUCCUGCUUCUCCUCCCCGC***UCGUGUCUUGUGUUGCAGCC***AGUGGAGCUGCCUA-----

fru-mir-187 -GUGGCCAUUUGGCCGGGCCAGGGGCUGCAACACAGGACAUGGGUCAUGCCUCUGCCCACCGC***UCGUGUCUUGUGUUGCAGCC***AGUGGAGCUG---------

tni-mir-187 -----------------------GGCUGCAACACAGGACAUGGGUCUUGCCUCUGCCCGCCGC***UCGUGUCUUGUGUUGCAGCC***AGUGG--------------

dre-mir-187-1 ---UGACCUGUGGCUGGGCCAGGGGCUGCAACACAGGACAUGGGAGCUGUCUCUCACUCCCGC***UCGUGUCUUGUGUUGCAGCC***AGUGGAACG----------

ccr-mir-187 -----ACCUGUGGCUGGGCCAGGGGCUGCAACACAGGACAUGGGAGCUGCCUCUCACUCCCGC**UCGUGUCUUGUGUUGCAGCCAGU**GGAACUGCUGCACUGU

********************* ** ** *****************************

hhi-mir-196 -----GCGGACUGUUGAGUGGUU**UAGGUAGUCUCAUGUUGUUGGG**-CUAAAUUAUUUCUCCCACAACACGAAACUGCCUUGAUUACCUCAGUA----------------

fru-mir-196a-1 --------AGCUGGAGCGUGGUU**UAGGUAGUUUCAUGUUGUUGGG**GAUGGCUUCCUGGCUCGGCAACAAGAAACUGCCUUGAUUACGUCAGUU----------------

ola-mir-196a UUGCGCGAAGCUGGAGCGUGGUU**UAGGUAGUUUCAUGUUGUUGG**GGUUGGCUUCCUGGCUCGGCAACAAGAAACUGCCUUGAUUACGUCAGUUCGUCUUCAUCAA----

tni-mir-196a-1 --------AGCUGGAGCGUGGUU**UAGGUAGUUUCAUGUUGUUGGG**GAUGGCUUCCUGGCUCGGCAACAAGAAACUGCCUUGAUUACGUCAGUUCGUCUUCAUCAAGGGC

dre-mir-196a-1 ---CGCGCGGCUGGUGCGUGGUU**UAGGUAGUUUCAUGUUGUUGGG**AUUGGCUUCCUGGCUCGACAACAAGAAACUGCCUUGAUUACGUCAGUUCGUCUUCAUCAAGGGC

ccr-mir-196a -----------UGGUGCGUGGUU**UAGGUAGUUUCAUGUUGUUGGG**AUUGGCUUCUUGGCUCGACAACAAGAAACUGCCUUGAUUACGUCAGUUC---------------

*** * ************** ************* * ** * * ***** ***************** *****

hhi-mir-199a --------------------GCCCGCCUG**CCCAGUGUUCAGACUACCUGUUC**AGGAAGUAGUGGUUGUACAGUAGUCUGCACAUUGGUUAGGCUGGCUGGGGAAGCACGGG

tni-mir-199-2 ----------------------CCGCCUG**CCCAGUGUUCAGACUACCUGUUC**AGGAAGUAGUGGUUGUACAGUAGUCUGCACAUUGGUUAGGCUG----------------

fru-mir-199-2 ----------------------CCGCCUG**CCCAGUGUUCAGACUACCUGUUC**AGGAAGUAGUGGUUGUACAGUAGUCUGCACAUUGGUUAGGCUG----------------

dre-mir-199-2 GGAGUUUUUGUGGACGCCCGUCCCGCCUG**CCCAGUGUUCAGACUACCUGUUC**AGGAAUUAGUGUUUG***UACAGUAGUCUGCACAUUGGUU***AGGCUGG---------------

ola-mir-199a-2 -------UCUCUAUCCCCCACCCCGCCUG**CCCAGUGUUCAGACUACCUGUU**CCUCCAGCUAUAACUGA***ACAGUAGUCUGCACAUUGGUUA***GGCUAGACUGGGAGCCACACA

ccr-mir-199 -----------------------CGUCAU**CCCAGUGUUCAGACUACCUGUUC**AGGAUCAUACUGGUGU***ACAGUAGUCUGCACAUUGGUU***AGAC------------------

pol-mir-199a-2 -----------------------------**CCCAGUGUUCAGACUACCUGUU**CAGGAAGUAGUGGUUGU***ACAGUAGUCUGCACAUUGGUUA***---------------------

*********************** **********************

hhi-mir-301 --GUUUUCAGCUAUUUGCAGGUGCUCUGACUUUGUUGCACUACUGUAUCAGACAG-CUAG**CAGUGCAAUAGUAUUGUCAAAGCA**UCUAGAAAACAGCAGUGAC-

ola-mir-301a ---GCCCGAGCUGUUGGCAGGU**GCUCUGACUUCAUUGCACUAC**UGUAUCAGACAU-CUAGUA***GUGCAAUAGUAUUGUCAAAGCA***UUU-GAAAACUGCAAAGAGC

dre-mir-301a ---------GCUGUUAACAGGUGCUCUGACUUCAUUGCACUACUGUAUUGGACAG-CUAG**CAGUGCAAUAGUAUUGUCAAAG**CGUCU-GAGAGCAGC-------

tni-mir-301 -------------AGGUCAGCUGCUCUGACAAUGUUGCACUACUGUACCAUCCAUUCUAG***CAGUGCAAUAGUAUUGUCAUAG***CAUUUGGCCU------------

fru-mir-301 -----------------CAGCUGCUUUGACAAUGUUGCACUACUGUACCAUCCAUUCUAG***CAGUGCAAUAGUAUUGUCAUAG***CAU-------------------

ccr-mir-301a ACAUCAAGUGCUGUUAACAGGUGCUCUGACUUCAUUGCACUACUGUAUUGGACAGCUAG**CAGUGCAAUAGUAUUGUCAAAGC**AUCUGAGAGCAGC---------

*** **** **** ************* ** **** ****************** *** *

hhi-mir-430a-1 AUCAAUUUAUCACCAAUAUUACCCUUGCACAAGCACCAACUUGAGUUUAGAAAACAG**UAAGUGCUAUUUGUUGGGGUAG**UAUUG-AUGA--

ola-mir-430a-3 -------GGACAUCAAGAUGACCUUGACACAGGCACUGACUCUUUCUUCUUUG-UAG**UAAGUGCUACUUGUUGGGGUA**AUUUUG-GUGUCC

dre-mir-430a-3 --------GUCACUAUCGGUACCCUCACAAAGGCACUGACUUGGAUGCUGUAAUUGG**UAAGUGCUAUUUGUUGGGGUAG**UUUCAAGUGAC-

** * *** * ** * **** *** ********** *********** * * **

hhi-mir-430a-2 GGUCCUACCUAUUACCCUAACUUGAGCAUUAACUUGUUUGUUGAAUGCAG**UAAGUGCUACUUGUUGGGGUAG**UUUUAAGUGAC-

dre-mir-430a-2 -GUCACUAUCGGUACCCUCACAAAGGCACUGACUUGGAUGCUGUAAUUGG**UAAGUGCUAUUUGUUGGGGUAG**UUUCAAGUGAC-

ola-mir-430a-2 GGACAUCAAGAUGACCUUGACACAGGCACUGACUC-UUUCUUCUUUGUAG**UAAGUGCUACUUGUUGGGGUA**AUUUUG-GUGUCC

ccr-mir-430 -----CUAUCGGUACCCUCACAAUGGCACUGACUUGUCUGUUGUAUUUGG**UAAGUGCUAUUUGUUGGGGUA**GUU----------

*** * ** *** * *** * * ********** *********** **

hhi-mir-430a-3 ACUAGGUGGUCACCAGCUUUACCCUAACACAAGCAAAGACUUGCCUUCUGAAAGCAG**UAAGUGCUAUUUGUUGGGGUAG**UGUUGC-UGA--

dre-mir-430a-1 --------GUCACUAUCGGUGCCCUCACAAAGGCACUGACUUGGAUGCUGCAUGUGG**UAAGUGCUAUUUGUUGGGGUAG**UUUCAAGUGAC-

ola-mir-430a-1 -------GGACAUAAAGAUGACCUUGACACAGGCACUGACUC-UUUCUUCUUUGUAG**UAAGUGCUACUUGUUGGGGUAU**UUUUGG-UGUCC

* ** * ** * *** * *** **** * * * ********** *********** * * **

hhi-mir-430b-1 GAAUCCAGCUCAGAUCACCUCAAAUGGAGCCACUGAUGAUUCUCUUCUUCA**UAAGUGCUUCUCUUUGGGGUUG**UCUUGGUUGGGAU--

dre-mir-430c-1 -------AUUAAGAUCACUUCAAACAGGAGCAUUGAUUUGUCCUUUGUUCA**UAAGUGCUUCUCUUUGGGGUAG**UUUUAAU--------

******* ***** * ** **** * * *** ******************** ** * *

hhi-mir-430b-2 ----CAACUCU-GAUCACCUCAAAUAGAGCCACUCAUGAUUACUGGCUUCA**UAAGUGCUUCUCUUUGGGGUUG**UCUUG--GUUG----

ola-mir-430d-1 GAAUCGCUUCGAGACCAUCUCAAACAGAGACACUGAUGGUU-UUGAUUUCC**UAAGUGCUUCUCUUUGGGGUUG**UCUCAAUGUUGGUUU

* ** ** ** ****** **** **** *** ** ** *** ************************* ****

hhi-mir-449 -UGUGGUGGGUGGCUGGAAGGCAGUGUCUUGUUAGCUGGUUGAUUGUGUGAGUG---CCAGCUUACCUGCUGCUGCCCUCCAACUGUCCACA--

mmu-mir-449a CUGUGUGUGAUGGCUUG---GCAGUGUAUUGUUAGCUGGUUGAGUAUGUGAGCGGCACCAGCUAACAUGCGACUGCUCUCCUAUUGCACACACA

**** * ***** * ******* *************** * ****** * ****** ** *** **** **** * ** ****

hhi-mir-723 -GGGCACGGAUAAAGGCAGUUUUGA-UGAUGUUACUUCUUUU--AUUUUAAGAAGACAUCAGAUAAAUCUGUGCUUAUCCUUGCUU---

dre-mir-723 UGCGUAGAGAUAAAGACAGUUUUAAAUGAUGUUACUUUUUUUCAAAUGGAGAAAGACAUCAAUUAAAUCUGUGCUUAUCUCUACAAGCA

* * * ******* ******* * *********** **** * * * ********* **************** * *

hhi-mir-728 GGCGCCCUGGGGAAAUGUAGUAGACUUUAAGUAUACGUGUGGAACCGGAGAGU**AUACUAAGUACACUACGUUUA**UCCAGGGG---

dre-mir-728 CAUCUUCUGAGGAAAUGUAGUAGACUAUAAGUAUACA-GUG-AACAUGAACGU**AUACUAAGUACACUACGUUUUC**UCAAGAGGUG

*** **************** ********* *** *** ** ********************** ** * *

hhi-mir-737 ---GUUCUACUCUUCUGUGGUUUUUUUAGGUUUUGAUUUUUGUAAACGUUGGAUGAGAAAAUCAAAACCUAAA-----------------------

dre-mir-737 CCACAGCUGCUGUGCUGUUGUUUUUUUAGGUUUUGAUUUUUGUGAAAUGUCGAUGAGAA**AAUCAAAACCUAAAGAAAAUA**CUGCGCAGAUAGAUGG

** ** * **** ************************ ** * **********************

hhi-mir-1788 GGAUAUACAGUCUUGUUUUCGA**GGC*UU*GUUUUAAGUUGCCUGCG**ACUUGUACACGGACU***CAGGCAGCUAAAGCAAGUCUGG***GACGCCACAGACACACCUACA

ola-mir-1788 -GAGCUUCAGUCCAGUUUUCGAGGCUUGUUUUAAGUUGCCUGCGGCUCGUACAUGGACA***CAGGCAGCUAAAGCAAGUC***UGGAACGCUGGAAACGCAC-----

dre-mir-1788 UCAACCACUGUCUUGUAUCCGA**GGCUUGUUUUAAGUUGCCUGCG**AUCUCUUAAUG-ACU***CAGGCAGCUAAAGCAAGUCUG***GGAGGCCAGAGACAACACGACA

* * *** ** * ************************* * * * ** ********************** * ** * **

**Partial precursor sequences**

hhi-let-7g ----------------------CAUUUCUG-GAUCGUGGGAGGAAUUAACGUCGUUCAUCAUUAUAUACAGUCUACUGUCUUUCACAGUUGUC----

fru-let-7g --------UGGGA**UGAGGUAGUAGUUUGUAUAGUU**UUAGGAUCACACCAGAUCUGGGAGAUAACUAUACAGUCUACUGUCUUUCCCA----------

tni-let-7g --------UGGGA**UGAGGUAGUAGUUUGUAUAGUU**UUAGGAUCACACCAGAUCUGGGAGAUAACUAUACAGUCUACUGUCUUUCCCA----------

dre-let-7g-2 GUGGACUGUGGGA**UGAGGUAGUAGUUUGUAUAGUU**UUAGGAUCACACCAGAUCUGGGAGAUAACUAUACAGUCUACUGUCUUUCCCACGGUUACCGC

ccr-let-7g ------UGUGGGA**UGAGGUAGUAGUUUGUAUAGUU**UUAGGAUCACACCAGAUCUGGGAGAUAACUAUACAGUCUACUGUCUUUCCCACGGUAA----

*** * * * *** * * ** * ******************** **

hhi-let-7i CCGGCCUCCUCGCUGUGUGGCCAGACACUGGC**UGAGGUAGUAGUUUGUGCUGUU**GGUUGGGUUGUGACACUGCCCGCUAUGGAGAUG----------------------------------------

fru-let-7i ---------------------------CUGGC**UGAGGUAGUAGUUUGUGCUGUU**GGUUGGGUUGUGACACUGCCCGCUAUGGAGAUGACUGCGCAAGCUACUGCCUUGCUA----------------

tni-let-7i ---------------------------CUGGC**UGAGGUAGUAGUUUGUGCUGUU**GGUUGGGUUGUGACACUGCCCGCUAUGGAGAUGACUGCGCAAGCUACUGCCUUGCUA----------------

dre-let-7i ----------------------GUGUACUGGC**UGAGGUAGUAGUUUGUGCUGUU**GGUUGGGAUGUGACAUUGCCCGUUAUGGAGAUGACUGCGCAAGCUACUCCCUUGCCAGUGCUG----------

ccr-let-7i ---------------UUUAGCCGUGUACUGGC**UGAGGUAGUAGUUUGUGCUGUU**GGUUGGGAUGUGACAUUGCCCGUUAUGGAGAUGACUGCGCAAGCUACUCCCUUGCCAGUGCUGGUUACACUCU

********************************** ******* ****** **********

hhi-mir-130 --------------------------------UACUGGACAUUGAAAUGAG**CAGUGCAAUAUUAAAAGGGCAU**UGGCUGAUGAAACACAGACCCA

fru-mir-130 UGUUGUUGUCCUCUGCCCUUUUUCUGUUGCACCACUGGACACUGAGAUGAG**CAGUGCAAUAUUAAAAGGGCAU**UGGCUG----------------

tni-mir-130 ---UGUUGUCCUCUGCCCUUUUUCUGUUGCACCACUGGACACUGAGAUGAG**CAGUGCAAUAUUAAAAGGGCAU**UGGC------------------

******** *** *******************************

hhi-mir-24b ----------------------------UACAGUUA--GAUGUUCACAAGCAC***UGGCUCAGUUCAGCAGGAACCG***GAGUUAGGCCCUUUAGUAAAC

fru-mir-24-1 GGGUUGUGCCCUCCU**GUGCCUACUGAACUGGUAUCAGU**GUCAUACUGGAAAAC***UGGCUCAGUUCAGCAGGAACAG***GAGU-----------------

tni-mir-24-1 ---------CUUCCUGUGCCUACUGAGCUGAUAU-GCAGUUGUACAGAACCAC***UGGCUCAGUUCAGCAGGAACAG***GAGUC----------------

dre-mir-24-1 ---ACCUGAGCUCCGGUGCCUUCUGAGCUGAUAUCA--GUUGUAGUAAAUCAC***UGGCUCAGUUCAGCAGGAACAGGA***GUGUGGCC-----------

ccr-mir-24 ------CUGGUCUCCUGUGCCUGCUGUGCUGAUAAUCAGUGGAUGGCUGUAGC**UGGCUCAGUUCAGCAGGAACAG***GG*GUCU---------------

* * * * * ********************* ** **

hhi-mir-216 --------UUGGUG**AAAUCCUCAGCUGGCAACUGUGA**GUCGUUCACUAGCUGC-------------------------------------

tni-mir-216a --------UUGGUG**AAAUC-UCAGCUGGCAACUGUGA**GUCGUUCACUAGCUGCUCUCACAAUGGCCUCUGGGAUUAUGCUAA--------

fru-mir-216a --------UUGGUA**AAAUC-UCAGCUGGCAACUGUGA**GUCGUUCACUAGCUGCUCUCACAAUGGCCUCUGGGAUUAUGCUAA--------

dre-mir-216a GCUGAUUUUUGGCA**UAAUC-UCAGCUGGCAACUGUGA**GUAGUGUUUUCAUCCCUCUCACAGGCGCUGCUGGGGUUCUGUCACACACAGCA

**** **** ******************* ** * *
