# Supplementary material for: Characterization of Novel Precursor miRNAs Using Next Generation Sequencing and Prediction of miRNA Targets in Atlantic Halibut
Source: PLoS One. 2013 Apr 23;8(4):e61378. doi: 10.1371/journal.pone.0061378 (PMC3634072; doi:10.1371/journal.pone.0061378)
Supplement: Dataset S3 — Hairpin structures predicted from Atlantic halibut sequenced genome data using srnaloop. The sequence centroid secondary structure and minimum free energy in kcal/mol are depicted. (DOC) [file pone.0061378.s004.doc]

**Dataset S3**

hairpin-n1 (hhi-mir-7788)

U AU U UAA AUG U - ACAU CA

UCAG G UUCAGAU U AG U U G GUUUAUUGU C

|||| | ||||||| | || | | | ||||||||| -21.60

GGUC U AAGUCUA A UU A A U CAAAUAAUA C

U -- C CAC CCG C G AUGU AA

hairpin-n3 (hhi-mir-7789)

UU CC UU UCC

GCUGC C CU UUCUGUUGC U

||||| | || ||||||||| -24.7

CGACG G GA GAGACGACG U

CU CA -C UCU

hairpin-n4 (hhi-mir-7790)

A C G GA AUUGUAUCA C

UU UUU CUACU UUUCCUU UUUCUU CCAUCUAGC C

|| ||| ||||| ||||||| |||||| ||||||||| -31.2

AA GAG GAUGA AAAGGAG AAAGAA GGUGGGUUG U

A A A -- --------- G

hairpin-n5 (hhi-mir-723)

C AG U -A -A CUUU

GGGCA GGAUAA GCAG UUUG UGAUGUU CUU U

||||| |||||| |||| |||| ||||||| ||| -34.9

UUCGU CCUAUU UGUC AAAU ACUACAG GAA A

U CG U AG AA UUUU

hairpin-n6 (hhi-mir-7791)

AAC GG ------ACU U AC AA U

AU UGG GCCAG G ACUGA UUUAA GUUAU G

|| ||| ||||| | ||||| ||||| ||||| -25

UG ACC CGGUC C UGACU AAAUU UAAUA A

-GC GA AAUUCAAUU U AU -A G

hairpin-n7 (hhi-mir-7792)

G U A CC C A A A ACUU

UU G AUCACUGA UGUG A UUGAG CG AAAUUGUU G G

|| | ||||||| ||| ||||| || |||||||| | C -15.7

AG C GUGACUA UGU AUUCA CG UUAAUGUG U U

A —A G -CU-C G U G AGUA

hairpin-n12 (hhi-mir-7793)

AG AG G A –- –- G GGUU

GU GGGU G UA GGUUAA GUUAA GUAG GUACC U

|| |||| | || |||||| ||||| |||| ||||| C -22.4

UG CUUG C AU CCAAUU CAAUU CAUU CAUGG C

GG GG - G GG GG G AUUUG

hairpin-n18 (hhi-mir-7794)

U A UGAAU CU GUAU

UUUGU C CUGCAGAG GUG CUAGAAUCCAG A

||||| | |||||||| ||| ||||||||||| A -34.6

AGACA G GAUGUUUC UAC GAUCUUGGGUC U

U G --UGU AC ACCC

hairpin-n20 (hhi-mir-449)

UG UGGC A -U CUU U UGAUU

UGUGG GG UGGA GGCAG GU GU AGCUGGU G

||||| || |||| ||||| || || ||||||| U -41.7

ACACC UC ACCU CCGUC CG CA UCGACCG G

UG ---A C GU -UC U UGAGU

hairpin-n22 (hhi-mir-430a-1)

---A C U -CA CA UGAG

UUU UCA CAAUAUUACCCU GCA AGCAC ACU UUUA

||| ||| |||||||||||| ||| ||||| ||| ||| A -34.3

AAA AGU GUUAUGAUGGGG UGU UCGUG UGA AAAA

CUUU A U UUA AA ---C

hairpin-n23 (hhi-mir-129-2)

C A CGU CGC G C -C UGU

GUU UG GGA CAGCA GGUU GGACCGUGAA A UUU C

||| || ||| ||||| |||| |||||||||| | ||| C -31.8

CGG AU CUU GUCGU UCGG UCUGGCGUUU U GGG U

A C --- --- G U CU UGUU

hairpin-n24 (hhi-mir-7795)

CA ---A AC GA --UG UGU

GA ACUU AAGGAGAC GU UGCUC UCAG G

|| |||| |||||||| || ||||| |||| -31.5

UU UGAA UUCCUCUG UA ACGAG GGUC U

UG AUAA AA AG UAUA CAA

hairpin-n29 (hhi-mir-7796)

-AA A AG ----ACC C

UUGACACAU UUG C CGGGUCUG CCGU U

||||||||| ||| | |||||||| |||| U -32.8

AACUGUGUG AAU G GCCCAGAC GGUA U

ACC C -A CCACUUA A

hairpin-n30 (hhi-mir-7641)

A C -------CG A

CUGA CA GCCCGAUCU UCCG U

|||| || ||||||||| |||| -25.7

GAUU GU CGGGCUGGG AGGC C

G C ACCAAUCAA U

**Precursors that did not meet miRBase guidelines**

hairpin-n8

CA UCACU UC UGAA AACGAC

UU CACACACAU CGUG CAAACAC AG U

|| ||||||||| |||| ||||||| || C -25.1

AA GUGUGUGUG GUAC GUUUGUG UC U

-C CCCGU CC UACA CACAGA

hairpin-n9

AGGUGA ---A --G UUUA G

GUGUUAGUGC GCU CUGCG GUGG UCAC U

|||||||||| ||| ||||| |||| |||| -31.6

CACAAUCACG CGA GACGU CACA AGUG U

-GUGUC AGAA AGU GUUG U

hairpin-n10

CAG CCAU ---CUC G A CC UG

UGGCUG CGG GUUUG U AGUG UGUCA GC C

|||||| ||| ||||| | |||| ||||| || -33.3

ACCGAC GCC CAAAC A UCAC ACGGU UG G

UAG ---A UUACCU G A AC CA

hairpin-n11

GG UCA -C -C C -G C C

CAGUG C GUGGAG GGU G UCACC GG GC G

||||| | |||||| ||| | ||||| || || A -35.2

GUCGC G CACCUU CCG C AGUGG CC CG C

AG CAC CA UC A AG A

hairpin-n13

AAAAC C GGUCAGUCAU GU U

CCAAAUG UAUU UUCAGUG CCACU GCG G

||||||| |||| ||||||| ||||| ||| -24.9

GGUUUGC GUGA AGGUCAC GGUGU CGU U

CAUGA C ---------A -G C

hairpin-n15

UGCC C A- - AAGUCU

UGAAGC AU UGAUGA C AAUGA U

::|||| || |||||| | ||||| U -12.6

GUUUCG UA ACUACU G UUACU C

UAAA U CA A AACUGU

hairpin-n17

A U C U U U C C - G AUCAC

UAAUGU U UA U GU G GAUGAA C A UAGA AAGU C

|||||| | || | || | |||||| | | |||| |||| C -13.7

GUUACA G GU A CA C CUACUU G U GUUU UUUA G

- U - C U U C U C G AUCCA

hairpin-n19

UCCC CGUGGGAC ---CAG AAAC GU

ACACG AC GGAUC AGGUGUG AACGU U

||||| || ||||| ||||||| ||||| -27.3

UGUGU UG CCUAG UCCACAU UUGCA U

U-UU -------A UCUUGG --CU UU

hairpin-n21

ACA CAG AG A CACUCA

GUGG GUUGGAGGGCAG C GU AGCUGG C

|||| |||||||||||| | || |||||| A -39.1

CACC CGACCUUCCGUC G CA UCGACC C

CAC ACA AA A AACUAA

hairpin-n25

A CC - GC C C U UC A ACGC

G GAU AGAGUGUC U U A ACGAG GA CUGCCG CA C

| ||| |||||||| | | | ||||| || |||||| || A -24

U UUA UCUCAUAG A A U UGUUU UU GACGGU GU A

G –- U AU A U - – G GUUAA

1. Kozomara A, Griffiths-Jones S (2011) miRBase: integrating microRNA annotation and deep-sequencing data. Nucleic Acids Research 39: D152-D157.
