# Supplementary material for: Characterization of Novel Precursor miRNAs Using Next Generation Sequencing and Prediction of miRNA Targets in Atlantic Halibut
Source: PLoS One. 2013 Apr 23;8(4):e61378. doi: 10.1371/journal.pone.0061378 (PMC3634072; doi:10.1371/journal.pone.0061378)
Supplement: Dataset S4 — Amplified fragments of Atlantic halibut miR-430 target genes using RACE-PCR. (PDF) [file pone.0061378.s005.pdf]

Dataset S4

> 3'end Salmo salar clone ssal-rgf-534-240 Collagen alpha-1I chain precursor putative mRNA
ACATGACATTGTTGTTTTTTTTTCTAGGGAAAAGCTCTAAATCCTTTTTTCTATGCATTCAA
AACTCATTCGGCTGCCATTGGTCCACATGCTTAAAACCACTCTACTGAAGACGGTGTTACT
CCAACTGAAACCAACCACAAGGACACTTTAAAAAGAATCGTTTGTTTCCACTGGCAACAA
GAATATAATATATACTTGTGTAAAATGTCCCCCTGGAGATTGAGAAAAAAAAAAAAAAAA
CCCCCACAAAAGAGACCCGGGGGGACTTTGGCCTTTTCCCCCCCTGGCGGAGGGAATTGA
AAAAAAAAGGGGATTTTACATTTTTGGGGGTAATAAGGAAGGGGGGGGCCTGTAACAAA
AAAACCCAAAAAAACCCTCCCAGGAAGGCCTTACAATCCCCTTTTTTTTAGAAGTTTTAAC
CAGTTCGGGAAAAAAATTAAGGGGGTTAAAACAAAAAAGCCCTTTTGGGGTTGAAGGGAA
AAATAAAAGCCCGCCAAACCGGAACTCTCGCCCCAAAGGGCTGCCAAACCTTTGGGGGCA
CCAAAATGGATTGGTGGCTTAGCAAAGCAATTGTTTTCCTTCTAAAGTGTGGGTTAGCCAA
CAAGGGCCAATCCAATGTTCCCCCCTGCAAAAATTCTTTCTCTGGTAAAAAAAAAAAGCA
GGGGCGTGGTCAAATTCTCATTCCCTGGCCGGGGGGCCAGGGGTGATTCTTTCCGGTGTGT
TGCCCGTGGGAAAAAAAAAAAAAACCCTCTCTTTTCTAAAAAAAAAAAAAAGCACCCTCTCT
CCCCCCCGTGTTGTTAGGGGTTTGGGGAAGTTTTTTTTTTCGCCACAAGGTTTTCTAAAAA
AAAATTTTTTGAAACAAAACTCTCTTTCTCGGTGTTTAAAAAAAAAAAAACCCGCAACCAA
ACGGCAGCACAGGTGTAAAAAAAAAAGACATCAGAAGGTTTTTTAAAAATATAGTTTGGGG
GGCGCTGTAATTCCCCCCCCTTTTTAAAATTTCCCTTCCTAACTGCCCCGGGGAAAAAAAA
AAAAAAAAAAAAAACAAACGTGACCCATGAGTAA

> 3' end Oreochromis niloticus CNDP2 mRNA for cytosolic nonspecific dipeptidase
CTACCGGCGGGGATTCGCCTTGCTGTCACGATACTCTACGTAACGGCATGCTGCGTCCATC
GGATCCTCCTGAAAAAGGGGCTCACTCCCAGAATGAGAAACTCAACAGATCCCACTATAT
TCAGGGAACCAAGATGCTGGGTGCATATTTCCATGAGGTCTCTCAGCTGGAATGAAGTAA
TGTCTCATCATGAAAAATGTAAAAGAGTGTCCATCCTCACTATCAGAGCTGTAGGACTTTG
TGGTATGATTTACCTTCCACAGTTTACGTTTTGTACACGCTGCCTCCTTATAATAAAAGTGT
TTCTTTGTTAAAAAAAAAAAAAAAAAAAAAAAAAAAAAAAAAAAAAAAAAAAAAAAA
AAAAAAAAAAAAAAAAAAAAAAAAAAAAAATAAAAAAAAAAATAAAAAAAAAAAAAAA
AAAAAAAAAAAAAAAAAAAAA

> 3'end osteonectin or SPARC mRNA
GCTGTCACGATACGCTACGCAACGGCGCTAATTGCAAATTATTTTTTATTTTGGAATGTTT
GAATAATGACTTTTCCTACCTATACCACTAAAAAGATTACAAAAGAGAAACGTGCACAGT
CTGTACTAACTTTGAATTCTCCTGTTGGTTGAACAAAACTCCCAGTAAAGTAAACTAAAGA
CCTCTGGTGTAAATGTATGTTGTGGACAATATTATTTTAGACGCTATGATTTGTTCAGGGCT
TTAAACGGAGAACAATTTGAAACGTTTTTATTGAGGAGACAAAAACTGTAAAGTCCAAAA
TAAAGTTTCTAAATAAATGCTCCCTCTGCTTGCTTTCTACAAAACCACGGTTTAGTTTTTGC
ACTCTGTTGGTGGTGGACTCCTTATTTTGTGTATTTTGGTCTTTGAACCTTTGACTACTGCTT
GTTCGTTGTTATTTTCCAAACAGAGTCCGTGTCCCGGTCCAGGACACGCGTGTACTGATAT
TTTGAAGCAATAAAGACGATATGTTGTTTTGGACGTGTCACTGAGGGGAAAATCTCGCAG
GTTGAGTGTTTTTCTCACTAATAATAACATGTTTCTACTGAGAGCACGACCAAAACTCTTC
CATGAATAAAAGACTATGAAAAAAAAAAAAAAAAAAAAAA
